# Mitochondrial topoisomerase 1 inhibition induces topological DNA damage and T cell dysfunction in patients with chronic viral infection

**DOI:** 10.3389/fcimb.2022.1026293

**Published:** 2022-11-03

**Authors:** Xindi Dang, Dechao Cao, Juan Zhao, Madison Schank, Sushant Khanal, Lam Ngoc Thao Nguyen, Xiao Y. Wu, Yi Zhang, Jinyu Zhang, Yong Jiang, Shunbin Ning, Ling Wang, Mohamed El Gazzar, Jonathan P. Moorman, Zhi Q. Yao

**Affiliations:** ^1^ Center of Excellence in Inflammation, Infectious Disease and Immunity, Quillen College of Medicine, East Tennessee State University, Johnson, TN, United States; ^2^ Division of Infectious, Inflammatory and Immunologic Diseases, Department of Internal Medicine, Quillen College of Medicine, ETSU, Johnson, TN, United States; ^3^ Hepatitis (HBV/HCV) and HIV Programs, James H. Quillen VA Medical Center, Department of Veterans Affairs, Johnson, TN, United States

**Keywords:** HCV, HIV, mitochondrial dysfunction, topoisomerase I, T cell dysregulation

## Abstract

T cells are crucial for controlling viral infections; however, the mechanisms that dampen their responses during viral infections remain incompletely understood. Here, we studied the role and mechanisms of mitochondrial topoisomerase 1 (Top1mt) inhibition in mitochondrial dysfunction and T cell dysregulation using CD4 T cells from patients infected with HCV or HIV and compared it with CD4 T cells from healthy individuals following treatment with Top1 inhibitor - camptothecin (CPT). We found that Top1mt protein levels and enzymatic activity are significantly decreased, along with Top1 cleavage complex (Top1cc) formation, in mitochondria of CD4 T cells from HCV- and HIV-infected patients. Notably, treatment of healthy CD4 T cells with CPT caused similar changes, including inhibition of Top1mt, accumulation of Top1cc in mitochondria, increase in PARP1 cleavage, and decrease in mtDNA copy numbers. These molecular changes resulted in mitochondrial dysfunction, T cell dysregulation, and programmed cell death through multiple signaling pathways, recapitulating the phenotype we detected in CD4 T cells from HCV- and HIV-infected patients. Moreover, treatment of CD4 T cells from HCV or HIV patients with CPT further increased cellular and mitochondrial reactive oxygen species (ROS) production and cell apoptosis, demonstrating a critical role for Top1 in preventing mtDNA damage and cell death. These results provide new insights into the molecular mechanisms underlying immune dysregulation during viral infection and indicate that Top1 inhibition during chronic HCV or HIV infection can induce mtDNA damage and T cell dysfunction. Thus, reconstituting Top1mt protein may restore the mtDNA topology and T cell functions in humans with chronic viral infection.

## Introduction

T cells play a critical role in controlling viral infections; however, the molecular mechanisms that dampen their responses during viral infections remain incompletely understood. We have recently reported that chronic viral (HCV, HIV) infection can cause T cell aging, as evidenced by the increases in the aging markers, telomeric and mitochondrial DNA damage, and mitochondrial dysfunction (Yao and Moorman, 2013; Shi et al., 2014; Li et al., 2015; Zhou et al., 2016; Nguyen et al., 2018; Zhao et al., 2018; Cao et al., 2019; Ji et al., 2019; Zhao et al., 2019; Dang, 2020; Khanal et al., 2020; Schank et al., 2020; Khanal et al., 2021; Nguyen L. N. et al., 2021; Nguyen L. N. N. T. et al., 2021; Schank et al., 2021; Schank et al., 2021; Wang et al., 2021; Zhao et al., 2021; Cao et al., 2022). Mitochondria are important cellular organelles responsible for energy production and oxidative metabolism and are a major source of reactive oxygen species (ROS). We have examined mitochondrial dysregulation in CD4 T cells during HCV or HIV infections and discovered that oxidative stress, caused by excess ROS production, induces mitochondrial injury and accelerates telomere erosion - a dual effect that can promote the T cell aging process and, eventually, cell death (Schank et al., 2020; Schank et al., 2021; Schank et al., 2021; Wang et al., 2021; Zhao et al., 2021). Thus, further investigation into how the mitochondrial machineries, especially mitochondrial DNA (mtDNA), are disrupted during chronic viral infections may provide new information regarding the mechanisms of CD4 T cell dysfunction and viral persistence (Schank et al., 2021; Zhao et al., 2021).

Human mtDNA sequencing revealed 16,569 base pairs of 37 small circular genes that encode 13 mitochondrial proteins - all of which are involved in the processes of oxidative phosphorylation (Anderson et al., 1981; Barshad et al., 2018). In essence, mtDNA damage disrupts the fine-tuned balance between ROS levels and ROS scavenging or antioxidative defenses by enzymes such as superoxide dismutase, catalase, and glutathione peroxidase, resulting in mitochondrial dysfunction, cell senescence, and programmed cell death (Barshad et al., 2018). Notably, mtDNA replication, transcription, and recombination can lead to topological tangles that must be untangled to ensure normal gene transactions and cellular functions (Champoux, 2001; Wang, 2002; Vos et al., 2011). To prevent or correct these topological derangements, topoisomerases - the enzymes that modulate the topology of DNA to prevent their entanglements - bind to and cut the double- or single-stranded DNA, allowing the DNA to be untangled. Failure to resolve the DNA knots can results in trapping of these enzymes at the target sites, thus generating topoisomerase cleavage complexes (Top1cc) and protein-linked DNA breaks (PDBs), and causing topological DNA damage and programmed cell death (Champoux, 2001; Pommier et al., 2006; Pommier, 2013).

Topoisomerases also play an important role in viral or bacterial DNA insertion into the host chromosomes. Notably, some chemotherapeutic drugs and fluoroquinolone antibiotics interfere with the activity of topoisomerases in bacteria or cancer cells and create topological DNA damage, which promotes cell death (Pouliot et al., 1999; Pommier et al., 2010; Guoa et al., 2014). Therefore, normal DNA topology is important for cellular functions, and its disruption can lead to topological DNA damage and cell death. We have previously reported that inhibition of topoisomerase 1 (Top1) and topoisomerase II alpha (Top2α) induces genomic DNA damage and T cell dysregulation during chronic viral (HCV, HBV, and HIV) infections (Ji et al., 2019; Dang, 2020). Notably, there are different isoforms of Top1 enzymes, e.g., a nuclear Top1 (Top1nc, ~100 kDa) and a mitochondrial Top1 (Top1mt, ~75 kDa), which are thought to function at their respective locations. Whether inhibition of these Top1 isoforms occurs in mitochondria (the major powerhouse and ROS source in cells) and thus causes mitochondrial dysfunction and T cell dysregulation during chronic viral infections have yet to be investigated.

In the present study, we assessed the levels of Top1 expression in mitochondria of CD4 T cells from patients with chronic HCV or HIV infection. We also recapitulated the phenotype of Top1 inhibition using healthy CD4 T cells treated with camptothecin (CPT) to investigate the role of Top1 in remodeling mitochondria and functions of CD4 T cells. We found that Top1 levels and enzymatic activity are remarkably inhibited in mitochondria of CD4 T cells from patients with HCV or HIV infection and in healthy CD4 T cells treated with CPT, thus implicating Top1 inhibition in promoting mitochondrial and T cell dysfunctions through regulating multiple cell death and metabolic pathways. These findings provide novel insights into the mechanism that disrupts mtDNA topology and its role in mitochondrial dysfunction and T cell dysregulation during chronic viral infection.

## Results

### Top1 protein levels and enzymatic activity are decreased in mitochondria of CD4 T cells in patients with HCV or HIV infection

**Figure 1:**
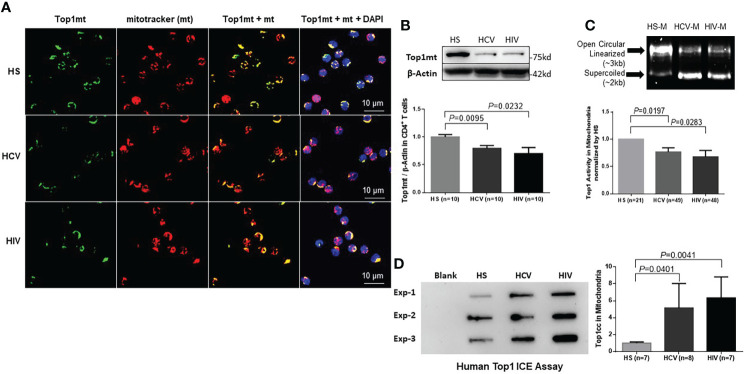
Top1mt expression and activity are inhibited in CD4 T cells during chronic viral infection. **(A)** Top1mt protein (green), MitoTracker (mt, red), and their colocalization (yellow) in mitochondria of CD4 T cells from HCV- and HIV-infected patients and healthy subjects (HS). The nucleus was stained with DAPI, and representative confocal microscopy images were merged. **(B)** Top1mt protein expression in CD4 T cells from HCV- and HIV-infected patients and HS. Representative images and summary data of Western blots are shown (n=10). The Top1mt band intensity was normalized by β-Actin. **(C)** Top1 activity in mitochondria of CD4 T cells from HCV- and HIV-infected patients versus HS. Representative images and summary data of Top1-mediated digestion of supercoiled DNA substrate are shown. **(D)** Top1cc was detected in mtDNA from CD4 T cells from HCV- and HIV-infected patients versus HS. Representative images and summary data of Top1cc band intensity (normalized to HS) are shown.

We have previously reported that chronic viral infection induces T cell aging and immune senescence due to accelerated telomeric DNA damage and mitochondrial dysfunction (Yao and Moorman, 2013; Shi et al., 2014; Li et al., 2015; Zhou et al., 2016; Nguyen et al., 2018; Zhao et al., 2018; Cao et al., 2019; Ji et al., 2019; Zhao et al., 2019; Dang, 2020; Khanal et al., 2020; Schank et al., 2020; Khanal et al., 2021; Nguyen L. N. et al., 2021; Nguyen L. N. N. T. et al., 2021; Schank et al., 2021; Schank et al., 2021; Wang et al., 2021; Zhao et al., 2021; Cao et al., 2022). Given the crucial role of DNA topology in maintaining mitochondrial integrity and cell function and viability (Champoux, 2001; Wang, 2002; Vos et al., 2011), here we sought to determine the levels of Top1 protein in the mitochondria of CD4 T cells from chronically HCV- or HIV-infected patients. To this end, we first determined whether Top1 protein is present in mitochondria of CD4 T cells. We performed immunostaining in CD4 T cells using an antibody specifically targeting Top1mt, followed by confocal microscopy. As shown in **Figure 1A**, Top1mt was primarily present in the mitochondria, as evidenced by the colocalization of Top1mt and mitotracker (mt, a mitochondrial marker) in the cytoplasm of these cells. We also used an antibody specifically targeting Top1nc for immunostaining and determined whether Top1nc protein can also localize in the mitochondria of CD4 T cells. Intriguingly, we found that Top1nc protein is also present in the mitochondria and co-localized with mt (**Figure S1A**). Notably, the fluorescent signals from Top1mt and Top1nc (green) as well as mt (red) and their co-localizations (yellow) were relatively lower in CD4 T cells from HCV or HIV patients compared with healthy subjects (HS) (**Figure 1A**, **Figure S1A**).

We then performed immunoblotting of Top1mt and Top1nc using whole cell lysates as well as fractionated mitochondrial and cytosolic extracts from CD4 T cells to determine the levels of Top1 proteins in these subcellular compartments. Mitochondrial heat shock protein 70 (mHsp70) is a mitochondrial resident protein which plays a critical role for protein translocation into the mitochondria. mHsp70 cooperates with Hsp10 and Hsp 60 and mediates essential functions for mitochondrial biogenesis, like protein folding and translocation into mitochondria (Goswami et al., 2010; Böttinger et al., 2015). mHsp70 and β-actin served as loading controls for mitochondria and cytosolic proteins, respectively. As shown in **Figure 1B**, CD4 T cell lysates from HCV- or HIV-infected patients exhibited significantly lower levels of Top1mt protein compared with the age-matched, healthy CD4 T cells. Additionally, these cells exhibited significantly lower levels of Top1nc in both mitochondrial (M) and cytosolic (C) compartments compared with the healthy CD4 T cells (**Figure S1B**). To determine the relationship between Top1 levels and CD4 T cell numbers in patients with HIV infection, we measured Top1nc protein levels in mitochondrial and cytosolic extracts from antiretroviral therapy (ART)-treated HIV patients with CD4 T cell counts > 500 cells/µL (defined as HIV immune responders/HIV-IRs) and CD4 T cell counts < 500 cells/µL (defined as HIV immune non-responders/HIV-INRs) (Zhao et al., 2021) and compared them with those from HS. As shown in **Figure S1C**, CD4 T cells from HIV-INRs exhibited the lowest Top1nc levels in both mitochondria and cytosol compared with CD4 T cells from HIV-IRs or HS, indicating that Top1nc protein levels correlate with CD4 T cell frequencies in HIV patients.

Human Top1 is a type 1B topoisomerase that can relax supercoiled DNA (Champoux, 2001). Thus, we used a plasmid (pHOT1)-based Top1 assay to measure Top1 enzymatic activity in CD4 T cells from HCV- and HIV-infected patients. We found that both mitochondrial (**Figure 1C**) and cytosolic extracts (**Figure S1D**) from CD4 T cells derived from these patients failed to completely relax the supercoiled plasmid DNA compared with CD4 T cells from HS, indicating a reduced level of Top1 enzymatic activity in the mitochondria and cytosol of CD4 T cells from patients with HCV or HIV infection.

To determine if the decrease in Top1 protein is due to its trapping at the mtDNA catalytic site, leading to Top1cc accumulation and then degradation, we used a monoclonal antibody that specifically targets covalent Top1-DNA complexes (but not free Top1 or DNA) and measured Top1cc by immunoblotting in mitochondria isolated from CD4 T cells (Patel et al., 2016). We found a significantly higher amounts of Top1cc in mtDNA of CD4 T cells from HCV or HIV patients compared with HS (**Figure 1D**).

Given our previous studies demonstrating premature CD4 T cell aging and dysfunction in chronic viral infections (Yao and Moorman, 2013; Shi et al., 2014; Li et al., 2015; Zhou et al., 2016; Nguyen et al., 2018; Zhao et al., 2018; Cao et al., 2019; Ji et al., 2019; Zhao et al., 2019; Dang, 2020; Khanal et al., 2020; Schank et al., 2020; Khanal et al., 2021; Nguyen L. N. et al., 2021; Nguyen L. N. N. T. et al., 2021; Schank et al., 2021; Schank et al., 2021; Wang et al., 2021; Zhao et al., 2021; Cao et al., 2022), the results described above suggest that topological DNA aberrancies (i.e., the decrease in Top1 protein and enzymatic activity and Top1cc accumulation) occur in the mitochondria of dysfunctional, senescent CD4 T cells during chronic HCV or HIV infections.

### Top1 inhibition by CPT treatment induces topological mtDNA damages in healthy CD4 T cells

**Figure 2:**
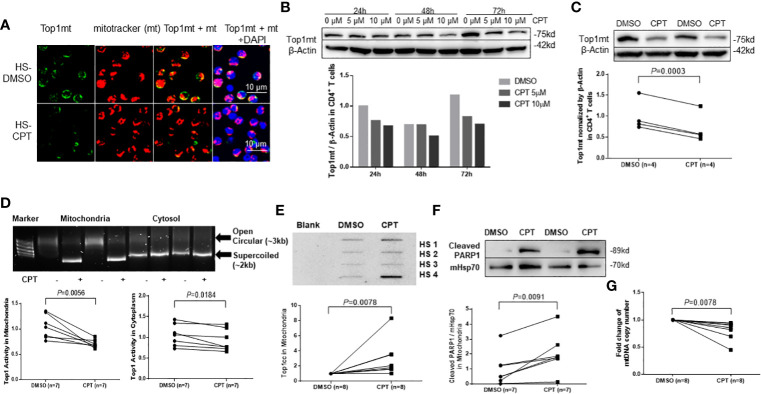
Top1 inhibition by CPT induces topological mtDNA damage in healthy CD4 T cells. **(A)** Top1mt protein (green), MitoTracker (mt, red), and their colocalization (yellow) in mitochondria in HS-CD4 T cells treated with DMSO or CPT (10 μM) for 24 h and observed by confocal microscopy. The nuclei were stained with DAPI, and representative confocal microscopy images were merged. **(B)** Immunoblotting of Top1mt in HS-CD4 T cells treated with CPT (5 μM or 10μM) or DMSO for 24 h, 48 h or 72 h. **(C)** Immunoblotting and summary data of Top1mt in HS-CD4 T cells treated with 10μM CPT or DMSO for 24 h. Representative images and summary data from independent experiments (n=4) are shown. **(D)** Top1 activity and summary data in mitochondria and cytosol in HS-CD4 T cells treated with CPT (5 μM) or DMSO for 24 h, determined by Top1 activity assay (n=7). **(E)** Immunoblotting and summary data of Top1cc in mtDNA from HS-CD4 T cells exposed to CPT (5 μM) or DMSO for 48 h (n=8). **(F)** Immunoblotting and summary data of PARP1 in mitochondria from HS-CD4 T cells treated to CPT (5 μM) or DMSO for 48 h (n=7). **(G)** mtDNA copy numbers relative to nuclear DNA were determined by real-time RT-PCR in HS-CD4 treated with CPT (5 μM) or DMSO for 24 h (n=8).

We have previously shown that healthy CD4 T cells treated with CPT, which inhibits Top1 protein expression, exhibit topological DNA damage and cell dysfunction, recapitulating the phenotype of CD4 T cells from HCV or HIV-infected patients (Ji et al., 2019). Mechanistically, CPT inhibits Top1 enzymatic activity by intercalating into the DNA at the catalytic site, leading to accumulation of the transcription-blocking Top1cc and PDB production, followed by degradation of the Top1cc and cell cytotoxicity (Pommier, 2006). To determine whether CPT treatment can lead to Top1 protein inhibition in mitochondria, we examined Top1 protein localization in CPT-treated CD4 T cells from HS using confocal microscopy. As seen in CD4 T cells from HCV- and HIV-infected patients, Top1mt (**Figure 2A**) and Top1nc (**Figure S2A**) proteins were primarily detected in the cytosol, co-localized with mitochondrial marker, and their levels decreased following CPT treatment compared with the dimethyl sulfoxide (DMSO) control treatment. We also employed immunoblotting to measure Top1mt and Top1nc protein levels in a mitochondrial extract from healthy CD4 T cells treated with CPT or DMSO. To do this, CD4 T cells from HS were treated with varying concentrations of CPT (0, 5, or 10 µM) for 24, 48, and 72 h. Significant inhibitions of Top1mt (**Figures 2B, C**) and Top1nc (**Figure S2B**) expression were observed following CPT treatment. Correspondingly, Top1 enzymatic activity was inhibited - more significantly in mitochondria than cytosol - in CD4 T cells treated with CPT compared with DMSO control, measured by the Top1 activity assay kit (**Figure 2D**). In addition, Top1cc accumulation in mtDNA was higher in CPT-treated healthy CD4 T cells (**Figure 2E**). These results indicate that inhibition of Top1 expression by CPT treatment in healthy CD4 T cells can recapitulate the mtDNA damage found in CD4 T cells from HCV- and HIV-infected patients (**Figure 1**), and thus CPT represents a reliable model to study the mechanism of Top1-mediated mtDNA topological aberrancies and their role in T cell dysregulation during viral infection.

Trapped Top1cc can cause PDBs, which are removed by tyrosyl-DNA phosphodiesterase-1 (TDP1) (Desai et al., 2003; El-Khamisy et al., 2005; Miao et al., 2006; Pommier et al., 2006; Capranico et al., 2007; Ashour et al., 2015). Top1cc excision by TDP1 requires Poly ADP-Ribose Polymerase 1 (PARP1) (Boulares et al., 1999; Pommier et al., 2006; Das et al., 2014). Specifically, PARP1 interacts with TDP1 and is recruited to the sites of Top1cc-induced PDBs to initiate the DNA repair process. Failure to complete this process leads to unrepaired DNA damage, PARP1 cleavage, and cell dysfunction or apoptosis. To determine whether CPT-induced mtDNA damage in healthy CD4 T cells triggers PARP1 cleavage, we measured the PARP1 level in mitochondrial extracts from CD4 T cells treated with CPT. As shown in **Figure 2F**, the level of cleaved PARP1 was significantly increased in mitochondria, suggesting its involvement in mtDNA damage and cell apoptosis. Top1cc trapping at the Top1-DNA interface leads to Top1 inhibition and subsequent degradation of Top1-PDB complexes and, ultimately, topological DNA damage (Goswami et al., 2010). To determine the consequences of Top1 inhibition, we measured mtDNA content (mtDNA copy numbers normalized to nuclear DNA) by qPCR and found that its level was significantly lower in healthy CD4 T cells following CPT treatment (**Figure 2G**). These CPT-induced changes are in line with our findings in CD4 T cells from HCV- and HIV-infected patients (Zhao et al., 2021; Schank et al., 2021).

### Top1 inhibition in healthy CD4 T cells induces mitochondrial oxidative stress and metabolic dysfunctions

**Figure 3:**
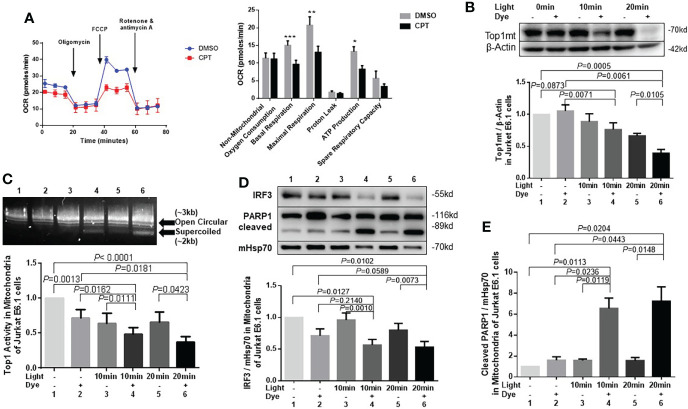
Top1 inhibition by CPT or cellular oxidative stress induces mitochondrial dysfunction in healthy CD4 T cells. **(A)** Oxygen consumption rate (OCR) for non-mitochondrial oxygen consumption, basal respiration, maximal respiration, proton leak, ATP production, and spare respiratory capacity in HS-CD4 T cells treated with CPT (5 μM) or DMSO for 48 h. Representative images and summary data from independent experiments are shown (n=7). **(B)** Western blot analysis of Top1mt levels (normalized to β-Actin) in E6-1 cells transfected with/mito-FAP-mCer3 cells and treated with or without MG2I dye plus light for 10-20 min (n=5). **(C)** Top1 enzymatic activity in mitochondria from E6-1/mito-FAP-mCer3 cells treated with or without MG2I dye plus light for 10 or 20 min (n=8). **(D)** Representative Western blots and summary data of IRF3, and representative data of total PARP1/cleaved PARP1 levels in mitochondria from E6-1/mito-FAP-mCer3 transfected cells treated with or without MG2I dye plus light for 10 or 20 min (n=6). **(E)** Summarized western blotting data of total PARP1, and cleaved PARP1 levels in mitochondria from E6-1/mito-FAP-mCer3 transfected cells treated with or without MG2I dye plus light for 10 or 20 min (n = 4) *p < 0.05, **p < 0.01, ***p < 0.001.

We next assessed whether CPT-induced Top1 inhibition affects T cell metabolic fitness. First, we evaluated mitochondrial function by measuring oxygen consumption rate (OCR) and ATP production in CPT-treated healthy CD4 T cells using Seahorse MitoStress Tests. In line with the presence of topological mtDNA damage, CPT-treated cells exhibited an impaired mitochondrial function, as evidenced by the significant decrease in the levels of basal and maximal OCR and ATP production compared with cells treated with DMSO (**Figure 3A**).

We have recently used a new chemoptogenetic method that can produce singlet oxygen (^1^O_2_) at specific organelles in CD4 T cells to show that oxidative stress promotes dual damage to mitochondria and telomeres in human T cells (Wang et al., 2021). To exclude the possibility of a non-specific or cytotoxic effect by CPT drug treatment, we employed this innovative chemoptogenetic tool to specifically target and induce oxidative stress in mitochondria, followed by examining mitochondrial Top1 protein level and enzymatic activity. This method employs a specific cellular protein tagging with fluorescent mCerulean (mCer) and fluorogenic-activating peptide (FAP) - to detect the expression of the fusion protein in a specific cellular compartment (Telmer et al., 2015; Fouquerel et al., 2019). FAPs have a high affinity to di-iodinated malachite green (MG2I, a photosensitizer dye), which produces singlet oxygen (^1^O_2_) only upon FAP binding and excitation with a light-emitting diode, thus triggering on-target oxidative damage without causing off-target injury (Telmer et al., 2015; He et al., 2016; Fouquerel et al., 2019). As shown in **Figure 3B** and **Figure S2C**, Top1mt and Top1nc levels were significantly reduced in a time-dependent manner in mitochondria of CD4 T cells following the exposure to light and MG2I dye compared with cells without these treatments. Also, Top1 enzymatic activity was significantly reduced in the mitochondria following the treatment with light plus dye compared with light or dye alone, or in cells without any treatment (**Figure 3C**). Interferon regulatory factor 3 (IRF3), a transcriptional factor, plays a key role in innate responses against viral infection. Previous studies show that IRF3 is phosphorylated and activated by the Serine/threonine-protein kinase TBK1 mediated by innate immune adaptor proteins MAVS, STING and TRIF, thereby driving the production of type-I interferons (Liu et al., 2015)IRF3 was significantly inhibited in mitochondria (**Figure 3D**), whereas cleaved PARP1, but not un-cleaved PARP1, was increased by this treatment (**Figure 3E**), indicating failed DNA damage repair and impaired cellular responses. These findings, in conjunction with our recent report showing mitochondrial dysfunction and cellular apoptosis following the same treatment (Wang et al., 2021), suggest that Top1 inhibition plays an important role in mitochondrial compromise and cell dysfunction.

### Top1 inhibition in healthy CD4 T cells by CPT induces cell dysfunctions *via* disrupting the cGAS-STING pathway

cGAS can sense DNA damage and trigger immune reactions *via* activating the adaptor protein STING on the endoplasmic reticulum (ER) surface (Ablasser et al., 2013; Cai et al., 2014; Li and Chen, 2018). STING then activates the transcription factors IRF3 and NF-κB, which translocate into the nucleus to turn on the transcription of inflammatory cytokines (Ahn et al., 2012; Chen et al., 2016). Thus, DNA damage can trigger cGAS-STING activation, and the cGAS-STING pathway can link DNA damage to inflammation, cell senescence and dysfunction (Ahn et al., 2012; Ablasser et al., 2013; Cai et al., 2014; Chen et al., 2016; Li and Chen, 2018).

**Figure 4:**
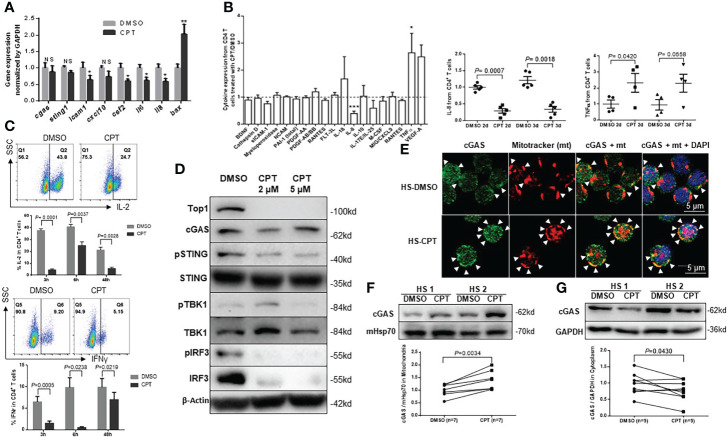
Top1 inhibition by CPT induces cell dysfunctions in healthy CD4 T cells *via* dysregulating the cGAS-STING pathway. **(A)** mRNA levels of cGAS/STING-related signaling genes were determined by real-time RT-qPCR in HS-CD4 T cells treated with CPT (5 μM) or DMSO for 24 h (n=9). **(B)** Cytokine array profile in cultured media of HS-CD4 T cells treated with CPT (5 μM) or DMSO for 48 h. Summary cytokine array data of IL-8 and TNFα in cultured media of HS-CD4 T cells treated with CPT (5 μM) or DMSO for 48 h and 72 h (n=5). **(C)** Representative dot plots and summary data of flow cytometry analysis of IL-2 and IFN-γ expression in HS-CD4 T cells treated with CPT (5 μM) or DMSO for 3 h, 6 h, and 48 h (n=4). **(D)** Protein levels of cGAS-STING-related signaling genes in HS-CD4 T cells treated with CPT (2 μM or 5 µM) or DMSO for 24 h, determined by Western blot. **(E)** Confocal microscopy images showing cGAS (green) and MitoTracker (mt, red) and their co-localization (yellow) in HS-CD4 T cells treated with CPT (5 μM) or DMSO for 24 h. **F, G**) Representative Western blots and summary data showing cGAS expression in the mitochondria (n=7) or cytosol (n=9) from HS-CD4 T cells treated with CPT (5 μM) or DMSO for 24 h. *p < 0.05, ** < 0.01, ***p < 0.001; ns, no significance.

To investigate the mechanism involved in the CPT-induced topological DNA damage and cellular dysfunction, we examined the expression levels of cGAS/STING-related signaling molecules using quantitative RT-PCR. As shown in **Figure 4A**, the levels of *cgas*, *sting1*, and *cxcl10* mRNA remained unchanged, whereas the levels of *icam1*, *csf2*, *il6*, and *il8* mRNA significantly decreased, and the levels of the pro-apoptotic *bax* mRNA significantly increased in healthy CD4 T cells treated with CPT compared with DMSO. We then measured the protein levels of these cytokines using supernatants of CD4 T cells exposed to CPT or DMSO for 2-3 days. As shown in **Figure 4B**, IL-8 was significantly down-regulated, whereas TNFα was significantly upregulated in the CPT-treated CD4 T cells. We also measured intracellular IL-2 and IFN-γ expression by flow cytometry in CD4 T cells exposed to CPT or DMSO for 3 h, 6 h, or 48 h in following stimulation with PMA/ionomycin/brefeldin A for 4 h. Notably, both cytokines were significantly down-regulated in the CPT-treated CD4 T cells (**Figure 4C**).

DNA damage dysregulates cell functions and triggers cell apoptosis by activating the cGAS/STING pathway (Ablasser et al., 2013; Cai et al., 2014; Li and Chen, 2018). Upon sensing nuclear or mitochondrial DNA damages, cGAS is recruited to the damaged DNA and activate STING *via* phosphorylation, leading to STING conformational changes to form transmembrane homodimers, which subsequently translocate from the ER to the Golgi apparatus (Ahn et al., 2012; Ablasser et al., 2013; Cai et al., 2014; Chen et al., 2016; Li and Chen, 2018). This process is thought to free STING to activate TANK-binding kinase 1 (TBK1) and IRF3 *via* a phosphorylation-dependent mechanism (Ahn et al., 2012; Ablasser et al., 2013; Cai et al., 2014; Chen et al., 2016; Li and Chen, 2018). Because cGAS and STING mRNA levels remained unchanged in CPT-treated healthy CD4 T cells (**Figure 4A**), we assessed the protein levels of cGAS-STING-related signaling molecules in CD4 T cells exposed to CPT (2-5 µM) or DMSO for 24 h using Western blotting. As shown in **Figure 4D**, Top1 protein levels were consistently decreased in CPT-treated CD4 T cells. Interestingly, while cGAS protein was slightly changed, phosphorylation of STING (pSTING^Ser366^) was significantly inhibited, and total STING levels remained unchanged in the CPT-treated cells. Correspondingly, the levels of pTBK1^Ser172^, but not total TBK1, decreased in the CPT-treated healthy CD4 T cells. In addition, the levels of pIRF3^Ser396^ and total IRF3 expression were decreased in the CPT-treated cells. These results demonstrated that CPT inhibits the STING-related signaling molecules in healthy CD4 T cells.

Because CPT treatment in healthy CD4 T cells induced significant mtDNA damage, we asked whether cGAS is recruited to the mitochondria upon CPT-induced mtDNA damage. Confocal microscopy examination revealed that, while the cGAS signal (green) was slightly affected by the CPT treatment, its co-localization (yellow) with the mitochondrial marker mt (red) was increased compared with the DMSO control treatment (**Figure 4E**). We then examined cGAS levels in the mitochondrial and cytosolic extracts. Interestingly, levels of cGAS protein were significantly higher in the mitochondrial fraction (**Figure 4F**), but decreased in the cytosolic fraction (**Figure 4G**) in CPT-treated healthy CD4 T cells, indicating cGAS translocation from the cytosol to mitochondria that are undergoing mtDNA damage. These novel findings - accumulation of cGAS in mitochondria with mtDNA damage and loss of cGAS in the cytosol - may explain why the STING-related signaling molecules were phosphorylated in the CPT-treated CD4 T cells.

### Top1 inhibition in HCV/HIV-CD4 T cells by CPT increases ROS production and enhances apoptosis

**Figure 5:**
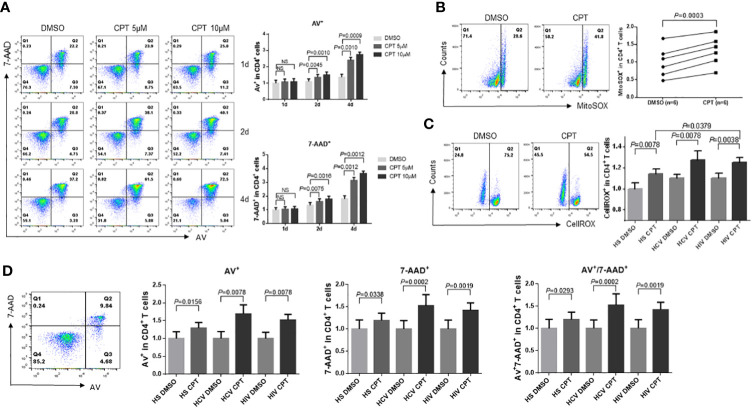
CD4 T cells from HCV- and HIV-infected patients are more susceptible to CPT-induced apoptosis than healthy CD4 T cells. **(A)** Representative dot plots and summary data of Av and 7-AAD staining in HS-CD4 T cells treated with CPT (5 or 10 μM) for 1 d, 2 d, and 4 d, determined by flow cytometry (n=6). **(B)** Representative dot plots and normalized summary data of MitoSOX production in CD4 T cells from HS treated with CPT (5 μM) or DMSO for 48 h (n=6). **(C)** Normalized cellular ROS production in CD4 T cells from HCV or HIV patients, and HS that were treated with CPT (5 or 10 μM) or DMSO for 48 h. Representative dot plots and summary data of CellROX was shown (n=8). **(D)** Flow cytometry gating strategy and summary normalized data of Av and 7-AAD staining in CD4 T cells from HS, HCV patients and HIV patients that were treated with CPT (5 μM) or DMSO for 24 h (n=8) ns, no significance.

To determine the consequences of topological mtDNA damage, we measured apoptosis in healthy CD4 T cells treated with CPT (5-10 µM) for 1, 2, and 4 days. Flow cytometry analysis showed increases in Annexin V (Av) and 7-Aminoactinomycin D (7-AAD) levels in a dose- and time-dependent manner in CPT-treated healthy CD4 T cells compared with DMSO control treatment (**Figure 5A**). We have previously shown that dysfunctional CD4 T cells with mitochondrial compromise produce more ROS, which promotes cell apoptosis during chronic HCV or HIV infections (Zhao et al., 2018; Zhao et al., 2019; Zhao et al., 2021; Schank et al., 2021). Thus, we used flow cytometry to measure mitochondrial ROS production in CD4 T cells from HCV- and HIV-infected patients and HS following exposure to CPT. Mitochondrial ROS (MitoSOX) production significantly increased in CPT-treated HS-CD4 T cells (**Figure 5B**), while total cellular ROS (CellROX) production significantly increased in CD4 T cells from HS as well as HCV or HIV patients following CPT treatment (**Figure 5C**). Additionally, CPT-treated CD4 T cells from HCV or HIV patients produced higher levels of cellular ROS compared with those from HS.

Since CD4 T cells from HCV or HIV patients are deficient in mitochondrial Top1 protein and enzymatic activity and exhibit more mtDNA damage, cell dysfunction, and ROS production, we asked whether these cells would be more susceptible to CPT-induced apoptosis. We thus assessed apoptosis in CPT-treated CD4 T cells from HCV or HIV patients and compared it with HS. As shown in **Figure 5D**, CPT-treated CD4 T cells from HCV or HIV patients exhibited higher rates of early (Av^+^), late (7-AAD^+^), and total (Av^+^ 7-AAD^+^) apoptotic cell death compared with HS-CD4 T cells. These results suggest that the topological mtDNA damage caused by Top1 inhibition, if left unrepaired, may promote cellular ROS production and trigger cell apoptosis and that CD4 T cells from HCV or HIV patients are more susceptible to cell death at least in part due to excess ROS production.

### Top1 inhibition in healthy CD4 T cells by CPT induces cell death *via* multiple signaling pathways

**Figure 6:**
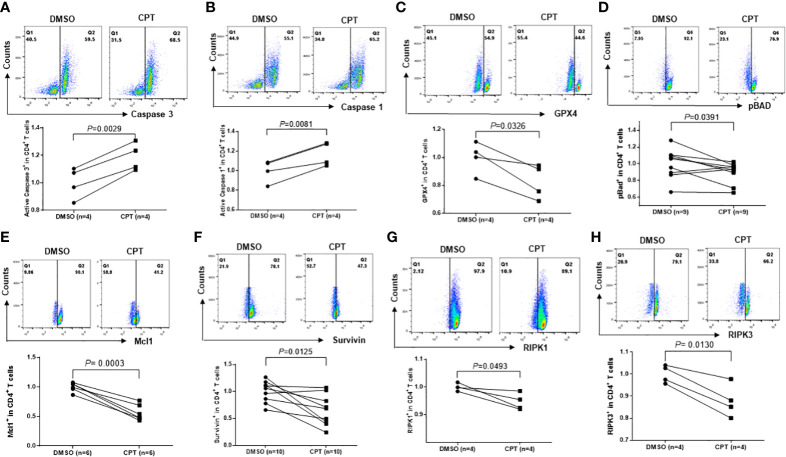
Top1 inhibition by CPT induces programmed cell death in healthy CD4 T cells *via* multiple cell death pathways. Healthy CD4 T cells were treated with CPT (5 μM) or DMSO for 24 h, followed by flow cytometry analysis of the expression levels of Caspase-3 **(A)**, Caspase-1 **(B)**, GPX4 **(C)**, pBAD **(D)**, Mcl1 **(E)**, Survivin **(F)**, RIPK1 **(G)**, and RIPK3 **(H)**.

CD4 T cell depletion is a hallmark of untreated HIV infection. We have previously reported that HIV infection induces CD4 T cell death through multiple death-related signaling pathways (Khanal et al., 2020; Cao et al., 2022). To elucidate the underlying mechanisms of CPT-induced CD4 T cell death, we examined alterations in cell death-related signaling molecules involved in cell apoptosis, pyroptosis, and ferroptosis using flow cytometry. Specifically, caspase-3 is activated by extrinsic and intrinsic pathways and plays a central role in cell apoptosis (Boatright and Salvesen, 2003). To determine whether apoptosis contributes to CD4 T cell depletion, we measured caspase-3 levels in healthy CD4 T cells exposed to CPT or DMSO for 24 h. Consistent with the increases in the levels of Av/7-AAD staining, caspase-3 levels were remarkably increased following CPT treatment compared with the DMSO control treatment (**Figure 6A**), indicating that apoptosis plays a role in the CD4 T cell depletion that is triggered by Top1 inhibition.

Pyroptosis is a form of programmed cell death that occurs due to inflammation caused by infection with intracellular pathogens, such as viruses (Fink and Cookson, 2005). Pyroptosis requires the activation of caspase-1, which increases the secretion of IL-1β and IL-18 cytokines. When immune cells recognize viral infection, they release pro-inflammatory cytokines and then die by pyroptosis. The cytokines released from pyroptotic cells promote injury of the surrounding bystander cells - a process that causes CD4 T cell depletion during HIV infection (Doitsh et al., 2014; Doitsh and Greene, 2016). Similar to caspase-3, caspase-1 was significantly upregulated in CPT-treated healthy CD4 T cells compared with the DMSO control (**Figure 6B**), suggesting that Top1 inhibition also promotes CD4 T cell depletion *via* pyroptosis.

Ferroptosis is an iron-dependent regulated cell death caused by the lethal accumulation of lipid-based reactive oxygen species (ROS) (Stockwell et al., 2017). Glutathione peroxidase 4 (GPX4), as a lipid repair enzyme that converts lipid peroxides into non-toxic lipid alcohols, is the primary cellular mechanism of protection against ferroptosis (Ref 53). Another parallel protective pathway against ferroptosis is the oxidoreductase FSP1, which functions as an oxidoreductase to reduce non-mitochondrial coenzyme Q 10 (CoQ), thereby generates a potent lipophilic antioxidant to suppress the distribution of lipid peroxides (Doll et al., 2019; Bersuker et al., 2019).

To determine whether ferroptosis plays a role in CD4 T cell death due to Top1 inhibition, we measured GPX4 expression in healthy CD4 T cells exposed to CPT or DMSO for 24 h. GPX4 levels were decreased, indicating increased ferroptosis following CPT treatment (**Figure 6C**). Taken together, these data demonstrate that Top1 deficiency in healthy CD4 T cells promotes programmed cell death *via* enhancing cell apoptosis, pyroptosis and ferroptosis.

To elucidate the mechanisms involved in the CD4 T cell programmed death following Top1 inhibition, we assessed the expression levels of pro- and anti-apoptotic proteins in CPT-treated healthy CD4 T cells using flow cytometry. Notably, BAX and BAD proteins are pro-apoptotic (Adefolaju et al., 2014), whereas phosphorylated BAD (pBAD^Ser136^) is anti-apoptotic - because canonic anti-apoptotic proteins such as BCL-2/BCL-XL form heterodimers with dephosphorylated BAD and triggers BAX-mediated apoptosis. When BAD is phosphorylated by AKT, BCL-2 is released and inhibits the BAX-triggered apoptosis (Scheid et al., 1999). We observed a decrease in pBAD protein in CPT-treated cells (**Figure 6D**). Moreover, we discovered that Survivin (survivin) and Mcl1 (two important anti-apoptotic proteins (Kuo et al., 2018; Warren et al., 2019)) were remarkably downregulated in CPT-treated cells (**Figures 6E, F**).

DNA damage responses (DDR) trigger specific signaling pathways that induce cell death, and the receptor-interacting protein kinase 1 and receptor-interacting serine/threonine-protein kinase 3 (RIPK1 and RIPK3) are crucial adaptor kinases at the crossroads of cell death (Festjens et al., 2007). Factors promoting survival and death compete with each other until one eventually dominates and dictates the cell fate (Festjens et al., 2007). We found that levels of both RIPK1 and RIPK3 were significantly decreased in healthy CD4 T cells following exposure to CPT compared with DMSO control treatment (**Figures 6G, H**). Taken together, the above results suggest that multiple signaling pathways are involved in the CD4 T cell death caused by Top1 inhibition.

## Discussion

CD4 T cells are crucial for controlling viral infections. However, chronic HCV or HIV infection blunts CD4 T cell functions and responses to vaccines (e.g., HBV vaccine) (Yao and Moorman, 2013). We have recently reported that CD4 T cells from patients with chronic HCV or HIV infection age prematurely or become senescent due to telomeric and mitochondrial DNA damage (Yao and Moorman, 2013; Shi et al., 2014; Li et al., 2015; Zhou et al., 2016; Nguyen et al., 2018; Zhao et al., 2018; Cao et al., 2019; Ji et al., 2019; Zhao et al., 2019; Dang, 2020; Khanal et al., 2020; Schank et al., 2020; Khanal et al., 2021; Nguyen L. N. et al., 2021; Nguyen L. N. N. T. et al., 2021; Schank et al., 2021; Schank et al., 2021; Wang et al., 2021; Zhao et al., 2021; Cao et al., 2022). The mechanisms underlying this DNA damage and the failure to repair it remain unknown. Because Top1 enzymatic activity is required to remove the DNA tangles that are generated during cell proliferation, and because Top1cc accumulates and becomes trapped during DNA transcription and causes Top1-linked PDBs (Champoux, 2001; Pommier et al., 2006; Pommier et al., 2010; Pommier, 2013; Guoa et al., 2014; Patel et al., 2016), we speculated that the topology mitochondrial DNA is altered due to Top1mt deficiency in mitochondria, leading to mitochondrial compromise and CD4 T cell dysfunction.

In the present study, we used CD4 T cells from patients with chronic HCV or HIV infection and healthy CD4 T cells, in which Top1 is inhibited by treatment with CPT, to examine Top1 inhibition and Top1cc formation in mitochondria and identify the molecular mechanisms underlying mtDNA damage and T cell dysfunction or cell death. We found that: 1) dysfunctional CD4 T cells from HCV- and HIV-infected patients exhibit lower levels of both Top1mt and Top1nc proteins and enzymatic activity, Top1cc accumulates in mitochondria, and the decrease in Top1 protein levels is associated with the degree of CD4 T cell depletion in HIV patients (HIV-INRs vs. HIV-IRs); 2) treatment of healthy CD4 T cells with CPT results in inhibition of Top1mt and Top1nc expression and enzymatic activity, Top1cc accumulation, and T cell dysfunction and apoptosis, recapitulating the phenotype we observed in HCV- and HIV-CD4 T cells and highlighting the role of Top1 in securing mtDNA integrity and maintaining T cell function and survival; 3) the mtDNA damage caused by Top1 inhibition in primary CD4 T cells leads to mitochondrial dysfunction - a process that could be recapitulated in CD4 T cells by oxidative injury induced by an innovative tool to specifically induce oxidative reactions at mitochondria; 4) these mtDNA damages lead to the translocation of cGAS from the cytosol into mitochondria, resulting in subsequent dephosphorylation or inactivation of STING signaling molecules and CD4 T cell dysfunction; 5) HCV- or HIV-CD4 T cells with Top1 inhibition are more susceptible to cell apoptosis, with excess mitochondrial and cellular ROS production; and 6) healthy CD4 T cells with Top1 inhibition undergo programmed death, triggered by multiple death signaling pathways. Taken together, these findings suggest that Top1 plays a pivotal role in preventing unwanted mtDNA damage and maintaining cell survival during chronic HCV or HIV infections.

**Figure 7:**
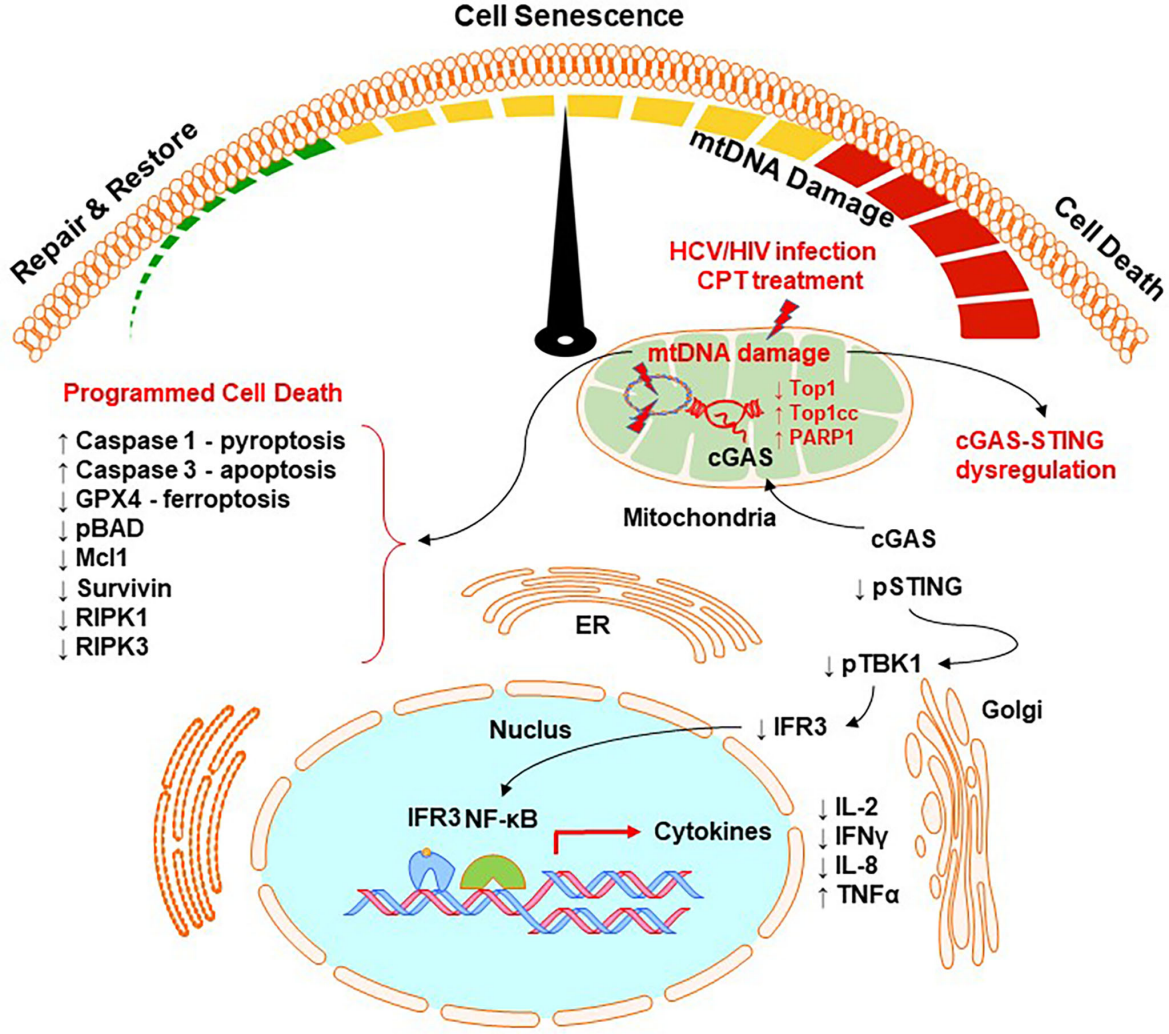
A working model for Top1-mediated topological DNA damage and T cell dysfunction. HCV/HIV infection or CPT treatment in healthy CD4 T cells can inhibit Top1 protein expression and enzymatic activity, leading to the accumulation of Top1cc and causing topological mtDNA damage, cell senescence, and programmed cell death through multiple signaling pathways. This topological mtDNA damage promotes cGAS translocation from cytosol to mitochondria, leading to dephosphorylation of STING, TBK1, IRF3, and thus inhibition of nuclear IRF3 and NF-κB-mediated gene transcriptions and cytokine expression. Topological mtDNA damage can also trigger multiple cell death or survival-related signaling pathways, leading to programmed cell death by apoptosis, pyroptosis, and ferroptosis. This regulatory cascade represents a novel molecular mechanism that underlies CD4 T cell senescence and dysfunction, which contribute to viral persistence and vaccine non-responsiveness during chronic HCV or HIV infection.

We found that the accumulation of topological mtDNA damage affects cell survival and functions. Based on our results, we propose a model, as depicted in **Figure 7**, where Top1 inhibition and Top1cc accumulation in mtDNA trigger Top1 proteolysis and Top1cc-linked PDB degradation, and thus topological mtDNA damage. Topological mtDNA damage leads to cGAS translocation from cytosol to mitochondria, resulting in dephosphorylation of STING, TBK1, IRF3 and thus inhibition of nuclear IRF3 and NF-κB-mediated gene transcription, cytokine expression, and cellular functions. This model is supported by our recent reports that deficiency of ATM (an important DNA damage and repair enzyme) during HCV or HIV infection promotes DNA damage, CD4 T cell dysfunction and apoptosis, likely due to the unrepaired DNA damage (Zhao et al., 2018; Zhao et al., 2019; Khanal et al., 2020; Cao et al., 2022).

While Topological DNA damage can occur in replicating T cells under physiological conditions, Top1cc arises under multiple pathological conditions, such as viral (HBV, HCV, HIV, EBV, or CMV) infections, treatment with antiviral or chemotherapeutic agents, chronic inflammation, and oxidative reactions. As such, PDBs triggered by Top1cc degradation and Top1 inhibition may have a profound impact on CD4 T cell senescence, leading to the remodeling of the mtDNA damage response and reprogramming of CD4 T cell metabolism and apoptosis. These events may arise from defective removal of Top1cc and deficiency of DNA damage repair by ATM during HCV or HIV infection (Zhao et al., 2018; Zhao et al., 2019). CD4 T cells are particularly prone and susceptible to Top1-mediated DNA damage and cell death due to the high rates of oxygen consumption, which enhances mitochondrial ROS production. In line with this, we have recently reported significant increases in ROS production in CD4 T cells from HCV or HIV patients (Schank et al., 2021; Schank et al., 2021).

Notably, ROS can promote Top1cc formation, causing DNA damage and ATM activation (Daroui et al., 2004; Guo et al., 2010). Although mitochondria are the cell powerhouses and a major source of ROS production, the concept that mtDNA is particularly susceptible to the effects of ROS generated through the respiratory chain remains debated (Vos et al., 2011). Indeed, mtDNA does not exhibit oxidative DNA damage more than nuclear DNA (Wang, 2002). In contrast, we have recently found that mtDNA is more stable than nuclear and telomeric DNA in response to selective oxidative stresses (Wang et al., 2021). Also, oxidative damage to DNA is repaired more efficiently in mitochondria than in the nucleus (Champoux, 2001). The mtDNA is packaged with protective proteins as the nuclear proteins that assemble the chromosomal structure (Pommier et al., 2006). Additionally, mitochondria possess an exclusive mechanism to maintain mtDNA integrity and stability *via* degrading the damaged mtDNA, followed by duplicating the repaired mtDNA. This mechanism does not exist in the nucleus and can only occur when multiple copies of mtDNA are present in mitochondria (Pommier, 2013).

The cGAS-STING pathway is usually activated *via* sensing the damaged DNA in cytosol (which leaks from the nucleus) and plays an important role in regulating cell function and survival (Ahn et al., 2012; Ablasser et al., 2013; Cai et al., 2014; Chen et al., 2016; Li and Chen, 2018). Intriguingly, our results indicate a unique ability of mtDNA (which are multiple, small circular DNAs and more stable relative to nuclear DNA) to recruit cGAS from the cytosol to mitochondria upon damage by Top1 inhibition, negatively affecting STING phosphorylation and its associated downstream signaling pathways in the ER and Golgi apparatus. In addition, we found that not only is Top1mt inhibited, but Top1nc is also located and inhibited in mitochondria in our cell model systems. This novel finding involving cCAS mitochondrial translocation and Top1nc mitochondrial localization and inhibition in primary CD4 T cells by CPT are significant. Given its novelty, we specifically used several experimental approaches to examine mitochondrial, cytosol, and nuclear fractions, and consistently detected Top1nc in the mitochondrial compartment. We believe that Top1nc is not exclusive to the cell nucleus and Top1mt is subject to CPT inhibition.

CPT induced a topological mtDNA damage event that is different from the nuclear DNA damage - which usually leaks into the cytosol to activate the cGAS-STING pathway and induce inflammatory responses (Ahn et al., 2012; Ablasser et al., 2013; Cai et al., 2014; Chen et al., 2016; Li and Chen, 2018). Topological mtDNA damage may also activate the ATM pathway (Guo et al., 2010; Zhao et al., 2018; Zhao et al., 2019; Khanal et al., 2020; Cao et al., 2022), and the outcome of mtDNA repair with mutation insertions may cause alterations in mtDNA encoding some mitochondrial proteins, which may affect the cell metabolism and/or fitness. If mtDNA damage remains unrepaired, it can activate multiple cell death signaling pathways, leading to programmed cell death. Hence, our study reveals a new model in which mtDNA topological damage contributes to the CD4 T cell aging, dysfunction, and apoptosis that occur during HCV or HIV infection.

Although study focused on HCV or HIV infections, our findings may be extended to other chronic viral infections, such as EBV and CMV. These viral infections lead to phenotypes similar to HCV or HIV infections; for example, T cell exhaustion and senescence. As observed in CD4 T cells, we expect to see similar changes in mtDNA topology in CD8 T cells, which often exhibit an even more exhausted and senescent phenotype. Importantly, topological DNA damage and repair may function as a double-edged sword, resulting in cell death during acute infection and immune tolerance during chronic infection. Nonetheless, this study reveals an important role for Top1 inhibition in promoting mitochondrial dysfunction and highlight the molecular aspects of immunomodulation in CD4 T cells that are caused by chronic HCV or HIV infections. This study also provides a potential strategy to restore impairment in mtDNA topology as a means to improve CD4 T cell functions during human viral diseases.

In summary, we showed that HCV or HIV infection inhibits Top1mt and Top1nc enzymes in CD4 T cells, leading to Top1cc entrapment in mtDNA, topological mtDNA damage, mitochondrial compromise, and T cell dysfunctions. Notably, we reproduced this mtDNA disruption and T cell dysregulation phenotypes in healthy CD4 T cells *ex vivo via* CPT-induced inhibition of Top1 proteins. Notably, Top1nc is only found in the nucleus in mouse fibroblasts (Dalla Rosa et al., 2014). In addition, CPT targets Top1nc rather than Top1mt in yeast cells (De la loza Díaz and Wellinger, 2009). Our results showed that Top1nc is also localized in mitochondria in human CD4 T cells. Thus far, the function of Top1nc in the mitochondria of human CD4 T cells is unclear. It is also unclear whether the inhibition of Top1mt we observed in healthy CD4 T cells is due to a direct effect of CPT on Top1mt or due to CPT targeting Top1nc and causing DDR and cell death, which indirectly impacts Top1mt. In addition, since CPT treatment induce both Top1mt and Top1nc inhibition, the overexpression of Top1mt in HIV or HCV patient CD4 T cells and knockdown of Top1nc in healthy CD4 T cells need to be done to separate their effects on regulating topological DNA damage and T cell dysfunction. Thus, our study uncovers a novel mechanism for immunomodulation by viral infections, i.e., dysregulation of mtDNA topology and T cell functions. Thus, restoring mtDNA topology during chronic viral infection may rescue CD4 T cell functions.

## Materials and methods

### Study subjects

The study protocol was approved by the joint institutional review board (IRB) of East Tennessee State University and James H. Quillen VA Medical Center (ETSU/VA IRB, Johnson City, TN). The study included three groups: 64 chronic HCV patients without antiretroviral therapy (ART); 83 HIV patients on ART) with undetectable HIV-RNA; and 76 age-matched healthy subjects (HS). HS blood samples were provided by BioIVT (Gray, TN) and were negative for HCV or HIV infections. The characteristics of the subjects enrolled in this study are described in **Table 1**.

**Table 1:** Demographic characteristics of the study participants.

Subjects	n	Age (Mean)	Gender (M/F)	Viral load and other characteristics
HCV	64	48	44/20	17,000-9,980,000 IU/ml, 44 GT1, 12 GT2, 8 GT3
HIV	83	43	72/11	All on ART with undetectable HIV-RNA
HS	76	41	56/20	All tested negative for HCV, HBV, and HIV

### Cell isolation and culture

Mononuclear cells (PBMCs) were isolated from peripheral blood by Ficoll density centrifugation (Cat# 45-001-750, GE Healthcare, Piscataway, NJ). CD4 T cells were isolated from PBMCs using the CD4 T cell negative selection kit (Cat# 130-096-533, Miltenyi Biotec, Auburn, CA). The cells were cultured in RPMI-1640 medium supplemented with 10% FBS (Cat# S11050H, Atlanta Biologicals, Flowery Branch, GA), 100 IU/ml penicillin, and 2 mM L-glutamine (Cat# 25-030-081, Thermo Fisher Scientific, Waltham, MA) and maintained at 37°C in 5% CO_2_ incubator.

### Confocal microscopy

1x10^6^ CD4 T cells were stained with fluorochrome conjugated antibodies using a previously described method (Wang et al., 2021). For Top1/MitoTracker staining, HS CD4 T cells were treated with 10μM CPT (Cat# SKU: TG4110, TopoGEN, Buena Vista, CO) or DMSO (Cat# D2650, Sigma-Aldrich, St. Louis, MO) for 2 days. For cGAS/MitoTracker staining, HS-CD4 T cells were treated with 5μM CPT for 2 days. The primary antibodies included MitoTracker (Cat# M22425, Thermo Fisher Scientific, Waltham, MA), Rabbit anti-cGAS (Cat# 79978, Cell Signaling Technology, Danvers, MA), Rabbit anti-Top1mt (Cat# PA5-51660, Thermo Fisher Scientific, Waltham, MA) and mouse anti-Top1nc conjugated with Alexa Fluor 488 (Cat# ab223421, Abcam, Cambridge, MA). The secondary antibody included anti-rabbit IgG-Alexa Fluor 488 (Ca# 4412, Cell Signaling Technology). The cells were mounted with DAPI Fluoromount-G (Cat# D1306, SouthernBiotech, Birmingham, AL) and visualized with a confocal laser scanning inverted microscope (Leica Confocal, Model TCS sp8, Germany).

### Isolation of mitochondrial and cytosolic proteins

The Qproteome mitochondria isolation kit (Cat# 37612, Qiagen, Germantown, MD) was used to isolate and purify mitochondrial and cytosolic extracts according to the manufacturer’s protocol. Briefly, approximately 5x10^6^ freshly isolated CD4 T cells were harvested and resuspended in 1 mL Lysis Buffer, followed by incubation on an end-over-end shaker for 10 min at 4°C. After centrifugation, the supernatant, which contained the cytosolic fraction was collected in a new tube and labeled as the 1^st^ part of the cytosolic extract. Next, the pellets were resuspended in a 1.5 mL disruption buffer and centrifuged to separate nuclei and cytosol. The supernatants were centrifuged at 6000 x g, for 10 min to isolate the mitochondria in the pellet and the supernatant as the 2^nd^ part of the cytosolic extract. To concentrate the combined cytosolic extract, an Amicon ultra-0.5 centrifugal filter unit (Cat# UFC503096, Millipore Sigma, St. Louis, MO) was used.

### Western blotting

CD4 T cells were treated with 2, 5, or 10 μM of CPT or DMSO control for different times and the mitochondrial and cytosolic extracts or whole cell lysates were lysed on ice in RIPA lysis buffer (Cat# BP-407, Boston BioProducts, Ashland, MA) in the presence of protease inhibitors (cOmplete™, Mini, EDTA-free Protease Inhibitor Cocktail. Cat# 11836170001, Sigma). Protein concentration was measured by the Pierce BCA protein assay kit (Cat# 23225, Thermo Fisher Scientific). To obtain enough mitochondrial proteins for immunoblotting, cells were pooled from multiple subjects. Mitochondrial and cytosolic extracts and whole lysates of CD4 T cells were separated by SDS-PAGE and transferred to polyvinylidene difluoride membranes. The membranes were blocked with 5% non-fat milk, 0.1% Tween-20 in Tris-buffered saline (TBS), and then incubated overnight with primary antibodies against Top1mt, Top1, pIRF3^Ser396^, IRF3, PARP1, cGAS, pSTING^Ser366^, STING, pTBK1Ser^172^, TBK1, GAPDH, β-actin (Human-Reactive STING Pathway Antibody Sampler Kit #38866, Cell Signaling), and mHsp70 (Cat# MABS1955-100uL, Millipore Sigma). The membranes were incubated with appropriate horseradish peroxide-conjugated secondary antibodies (Anti-rabbit IgG, HRP-linked Antibody #7074, Anti-mouse IgG, HRP-linked Antibody #7076, Cell Signaling Technology, Danvers, MA), and the protein bands were developed and visualized with the Amersham ECL prime western blotting detection reagent (Cat# 45-002-401, GE Healthcare Bio-Sciences, Pittsburgh, PA). The protein bands were captured and quantified by the Chemi DocTM MP imaging system (Bio-Rad, Hercules, CA).

### Top1 activity assay

The enzymatic activity of Top1 was measured using the Top1 activity assay kit (Cat# TG1015–1, TopoGEN, Buena Vista, CO). Briefly, mitochondrial and cytosolic extracts were isolated from patients and HS CD4 T cells as described above. Mitochondrial or cytosolic proteins were mixed with plasmid DNA substrate and reaction buffer for 30 min at 37°C, diluted with Stopping Buffer containing protein loading dye, and electrophoresed on a 1% agarose gel for 2 h at 5~10 V/cm. The supercoiled DNA bands were visualized with a UV transilluminator and quantified by densitometry.

### Top1cc detection

Top1cc was detected using the Human Topoisomerase ICE assay kit (Cat# TG1020–1, TopoGEN). The mtDNA purification protocol was modified by combining the ICE assay kit and the PureLink Genomic DNA Mini kit (Cat# K182001, Thermo Fisher Scientific). Briefly, mtDNA was isolated from mitochondrial extracts using extraction buffer from the ICE assay kit and then purified using purification columns from the PureLink Genomic DNA Mini kit. The DNA samples were loaded using a vacuum pump onto an NC membrane, which was incubated with primary anti-Top1cc antibody from the ICE assay kit, followed by Western blotting.

### Flow cytometry

Intracellular IL-2 and IFN-γ cytokine production, cell apoptosis assay for Av/7-AAD expression, cell death signaling molecules (Caspase-1, Caspase-3, GPX4, pBAD^S136^, Mcl1, Survivin, RIPK1, and RIPK3), mitochondrial ROS production (MitoSOX™ Red Mitochondrial Superoxide Indicator. Cat# M36008) and cellular ROS production (CellROX™ Green Reagent, for oxidative stress detection. Cat# C10444) was determined by flow cytometry base on the guideline of the products, as described previously (Wang et al., 2021; Cao et al., 2022). For IL-2 and IFN-γ cytokine production, antibodies anti-IL-2- FITC (Cat# 500304) and anti-IFN-γ-PE (Cat# 12-7029-42) antibodies (Biolegend, San Diego, CA) were used to stain purified HS CD4 T cells treated with DMSO or CPT for 3h, 6h, and 2 days. For apoptosis analysis, the cells were washed with DPBS and stained using PE Annexin V apoptosis detection kit I (Cat# 559763, BD Biosciences, San Jose, CA) in a 1X binding buffer according to the manufacturer’s protocol. Controls for these assays included unstained cells, isotype control antibodies, and single positive staining, which were used for gating and compensation. Samples were analyzed with a BD AccuriC6 Plus flow cytometer and FlowJo V10 software.

### Cytokine array

CD4 T cells isolated from 5 HS were cultured and treated with CPT (5 µM) or DMSO for 2 or 3 days. Approximately, 250 µl of the culture supernatants were collected for cytokine expression analysis. Briefly, Human Cytokine 48-Plex Discovery Assay (Cat# HD48) and Human Supplemental Biomarker 10-Plex Discovery Assay (Cat# HDHSB10) were performed by Eve Technologies (Calgary, AB Canada). Data were normalized to the expression levels in the DMSO-treated cells.

### Seahorse XFp Cell Mito stress test

Seahorse XFp Cell Mito stress test (Cat# 103010-100, Agilent Technologies, Santa Clara, CA) was performed according to the manufacturer’s protocol using an XFp instrument. CD4 T cells from healthy subjects were purified from PBMCs, cultured in complete RPMI-1640 medium with 10% FBS, and treated with 5 μM CPT or DMSO for 2 days. One day prior to the assay, Seahorse mini cartridges were hydrated overnight in a non-CO_2_ incubator. On the day of the assay, the treated cells were seeded onto mini culture plates pre-coated for 1 h with poly-D-lysine (Thermo Fisher Scientific). Approximately 100,000 cells per well were cultured in Seahorse XF RPMI assay medium supplemented with 1.0 mM of glucose, 100 µM of pyruvate, and 1.0 mM of glutamine. The following inhibitors from the Cell Mito stress test kit were added to the culture media in this order: 2.0 μM of Oligomycin, 1.5 μM of FCCP, and 2.0 μM of Rotenone/Antimycin A, and the related three sequential measurements were recorded. Data analysis was performed using the Seahorse Wave software and the Seahorse Mito stress test report generator.

### Singlet oxygen induction in E6-1 cell line

The protocol for singlet oxygen induction in E6-1 cells and the treatment with light and dye was carried out as we previously described (Wang et al., 2021). Top1, IRF3, and total and cleaved PARP1 protein levels were measured by Western blot, and Top1 enzymatic activity was determined by the Top1 activity assay as described above.

### Real-time qPCR

Mitochondrial DNA (mtDNA) and nuclear DNA (nuDNA) contents in genomic DNA were determined by real-time qPCR according to previously described methods (Wang et al., 2021). Briefly, genomic DNA was extracted from CD4 T cells using the PureLink Genomic DNA isolation kit (Thermo Fisher Scientific). DNA concentration was measured by the Synergy H1 BioTek plate reader. The primers used for mitochondrial and nuclear DNA PCR are shown in **Table 2**. Approximately 25 ng of genomic DNA was used for the PCR reaction. The PCR cycling conditions were: 1 cycle at 50 °C for 2 min, 1 cycle at 95 °C for 10 min, and 40 cycles at 95 °C for 15 s and 62 °C for 60 s. The averages of mtDNA and nucDNA Cq values from triplicate reactions were calculated. The mitochondrial DNA content was determined using the following equations: ΔCq = (nucDNA Cq – mtDNA Cq); relative mitochondrial DNA content = 2 × 2ΔCq 30 (Rooney et al., 2015).

**Table 2:** PCR primers used in this study.

mtDNA tRNALeu	5′-CACCCAAGAACAGGGTTTGT-3′	5′-TGGCCATGGGTATGTTGTTA-3′
nuDNA β2-microglobulin	5′-TGCTGTCTCCATGTTTGATGTATCT3′	5′- TCTCTGCTCCCCACCTCTAAGT3′
*gapdh*	5′-TGACGAAAGCTGATATGCAA -3′	5′-GAGCAGGAGAAACTCCATTT-3′
*cgas*	5′-AAGGATAGCCGCCATGTTTCT-3′	5′-TGGCTTTCAGCAAAAGTTAGG-3′
*sting1*	5′-AGCATTACAACAACCTGCTACG-3′	5′-GTTGGGGTCAGCCATACTCAG-3′
*il6*	5′-GGTACATCCTCGACGGCATCT-3′	5′-GTGCCTCTTTGCTGCTTTCAC-3′
*il8*	5′-AAGGAAAACTGGGTGCAGAG-3′	5′-ATTGCATCTGGCAACCCTAC-3′
*icam1*	5′-AGCTTCGTGTCCTGTATGGC-3′	5′-TTTTCTGGCCACGTCCAGTT-3′
*cxcl10*	5′-AGTGGCATTCAAGGAGTACC-3′	5′-TGATGGCCTTCGATTCTGGA-3′
*csf2*	5′-GCCAGCCACTACAAGCAGCAC-3′	5′CAAAGGGGATGACAAGCAGAAAG3′
*ifnα*	5′GTGAGGAAATACTTCCAAAGAATCAC3′	5′-TCTCATGATTTCTGCTCTGACAA-3′
*ifi16*	5′-GAAGTGCCAGCGTAACTCCTA-3′	5′-TACCTCAAACACCCCATTCAC-3′
*bax*	5′-TGGAGCTGCAGAGGATGATTG-3′	5′-CCCAGTTGAAGTTGCCGTCAG-3′

The expression of pro-apoptosis genes and the cGAS-STING-related genes was determined by real-time RT-qPCR. Total RNA was extracted from ~2 × 10^6^ CD4 T cells treated with CPT for 48 h using the PureLink RNA Mini kit (Cat# 12183018A, Invitrogen), and cDNA was synthesized using the High Capacity cDNA Reverse Transcription kit (Cat# 4368814, Applied Biosystems, Foster City, CA) according to the manufacturer’s instructions. The PCR reactions were performed in triplicate. The PCR primer sequences are shown in **Table 2**. The PCR conditions were the same as described above. Gene expression was calculated using the 2^−ΔΔct^ method, normalized to GAPDH level, and is presented as fold change.

### Statistics

The data were analyzed using Prism 7 software and are presented as mean ± SEM. Differences between two groups were analyzed by independent Student’s t-test or paired t-test and by one-way ANOVA for multiple groups. P-values of <0.05 (or *), <0.01 (or **) and <0.001 (or ***) were considered statistically significant and very significant, respectively.

## Data availability statement

The original contributions presented in the study are included in the article/**Supplementary Material**. Further inquiries can be directed to the corresponding author.

## Ethics statement

The study protocol was approved by the joint institutional review board (IRB) of East Tennessee State University and James H. Quillen VA Medical Center (ETSU/VA IRB, Johnson City, TN). Written informed consent was obtained from all subjects.

## Author contributions

XD and DC performed most of the experiments; JZ, MS, LN, and SK, performed some experiments. XW and YZ provided technical support. JYZ, SN, LW, ME, and JM offered intellectual input for troubleshooting and discussed the findings. ZY supervised the project and wrote the manuscript with the help of all other authors. All authors contributed to the article and approved the submitted version.

## Funding

This work was supported by National Institutes of Health grants R01AI114748, R21AI138598, R21AI157909, and R15AG069544 (to ZY); VA Merit Review Awards 1I01BX004281 (to ZY) and 5I01BX005428 (to JM); DoD Award PR170067 (to ZY). This publication is the result of work supported with resources and the use of facilities at the James H. Quillen Veterans Affairs Medical Center. The contents in this publication do not represent the views of the Department of Veterans Affairs or the United States Government.

## Conflict of interest

The authors declare that the research was conducted in the absence of any commercial or financial relationships that could be construed as a potential conflict of interest.

## Publisher’s note

All claims expressed in this article are solely those of the authors and do not necessarily represent those of their affiliated organizations, or those of the publisher, the editors and the reviewers. Any product that may be evaluated in this article, or claim that may be made by its manufacturer, is not guaranteed or endorsed by the publisher.

## Abbreviations

7-AAD, 7-Aminoactinomycin D; AIDS, Acquired immune deficiency syndrome; AKT, Protein Kinase B; ATM, Ataxia telangiectasia mutated; ATP, Adenosine Triphosphate; Av, Annexin V; BAD, BCL-2-associated death promoter; BAX, BCL-2-associated X protein; BCL-2, B-cell lymphoma 2; BCL-XL, B-cell lymphoma-extra-large; bp, Base pairs; cGAS, Cyclic GMP-AMP synthase; CMV, Cytomegalovirus; CPT, Camptothecin; CSF2, Granulocyte-macrophage colony-stimulating factor; CXCL10, C-X-C motif chemokine ligand 10; DAPI, 4′,6-diamidino-2-phenylindole; DDR, DNA damage response; DMSO, Dimethyl sulfoxide; EBV, Epstein-Barr virus; FAP, Fluorogenic-activating peptide; GAPDH, Glyceraldehyde 3-phosphate dehydrogenase; GPX4, Glutathione peroxidase 4; HBV, Hepatitis B virus; HCV, Hepatitis C virus; HIV, Human immunodeficiency virus; HIV-INRs, HIV immune non-responders; HIV-IRs, HIV immune responders; HS, Healthy subjects; ICAM-1, Intercellular Adhesion Molecule 1; IFI16, Gamma-interferon-inducible protein; IFN-γ, Interferon gamma; IL-18, Interleukin-18; IL-1β, Interleukin 1 beta; IL-2, Interleukin-2; IL6, Interleukin 6; IL8, Interleukin 8; IRB, Institutional review board; IRF3, Interferon regulatory factor 3; Mcl1, Induced myeloid leukemia cell differentiation protein; MG2I, Di-iodinated malachite green; mHsp70, Mitochondrial 70 kilodalton heat shock proteins; mtDNA, Mitochondrial DNA; NF-κB, Nuclear factor kappa-light-chain-enhancer of activated B cells; NS, No significance; OCR, Oxygen consumption rate; PARP1, Poly ADP-Ribose Polymerase 1; PBMC, Peripheral blood mononuclear cell; PDBs, Protein-DNA breaks; POT1, Protection of telomeres protein 1; RIPK1, Receptor-interacting protein; RIPK3, Receptor-interacting serine/threonine-protein kinase 3; ROS, Reactive oxygen species; RT-PCR, Reverse transcription polymerase chain reaction; STING, Stimulator of interferon genes; TBK1, TANK-binding kinase 1; TBS, Tris buffered saline; TDP1, Tyrosyl-DNA phosphodiesterase 1; TNFα, Tumor necrosis factor alpha; TOPcc, Topoisomerase-DNA covalent complexes; Top1 or Top1nc, DNA topoisomerase 1; Top1cc, Top1 enzyme–DNA covalent complexes; Top1mt, DNA topoisomerase I mitochondrial; TOP2α, DNA topoisomerase 2-alpha.

## Glossary

**Table:**

7-AAD	7-Aminoactinomycin D
AIDS	Acquired immune deficiency syndrome
AKT	Protein Kinase B
ATM	Ataxia telangiectasia mutated
ATP	Adenosine Triphosphate
Av	Annexin V
BAD	BCL-2-associated death promoter
BAX	BCL-2-associated X protein
BCL-2	B-cell lymphoma 2
BCL-XL	B-cell lymphoma-extra-large
bp	Base pairs
cGAS	Cyclic GMP-AMP synthase
CMV	Cytomegalovirus
CPT	Camptothecin;
CSF2	Granulocyte-macrophage colony-stimulating factor
CXCL10	C-X-C motif chemokine ligand 10
DAPI	4′6-diamidino-2-phenylindole
DDR	DNA damage response
DMSO	Dimethyl sulfoxide
EBV	Epstein-Barr virus
FAP	Fluorogenic-activating peptide
GAPDH	Glyceraldehyde 3-phosphate dehydrogenase
GPX4	Glutathione peroxidase 4
HBV	Hepatitis B virus
HCV	Hepatitis C virus
HIV	Human immunodeficiency virus
HIV-INRs	HIV immune non-responders
HIV-IRs	HIV immune responders
HS	Healthy subjects
ICAM-1	Intercellular Adhesion Molecule 1
IFI16	Gamma-interferon-inducible protein
IFN-g	Interferon gamma
IL-18	Interleukin-18
IL-1b	Interleukin 1 beta
IL-2	Interleukin-2
IL6	Interleukin 6
IL8	Interleukin 8
IRB	Institutional review board
IRF3	Interferon regulatory factor 3
Mcl1	Induced myeloid leukemia cell differentiation protein
MG2I	Di-iodinated malachite green
mHsp70	Mitochondrial 70 kilodalton heat shock proteins
mtDNA	Mitochondrial DNA
NF-kB	Nuclear factor kappa-light-chain-enhancer of activated B cells;
NS	No significance
OCR	Oxygen consumption rate
PARP1	Poly ADPRibose Polymerase 1
PBMC	Peripheral blood mononuclear cell
PDBs	Protein-DNA breaks
POT1	Protection of telomeres protein 1
RIPK1	Receptor-interacting protein
RIPK3	Receptor-interacting serine/threonineprotein kinase 3
ROS	Reactive oxygen species
RT-PCR	Reverse transcription polymerase chain reaction
STING	Stimulator of interferon genes
TBK1	TANK-binding kinase 1
TBS	Tris buffered saline
TDP1	Tyrosyl-DNA phosphodiesterase 1
TNFα	Tumor necrosis factor alpha;
TOPcc	Topoisomerase-DNA covalent complexes
Top1 or Top1nc	DNA topoisomerase 1
Top1cc	Top1 enzyme–DNA covalent complexes
Top1mt	DNA topoisomerase I mitochondrial
Top2α	DNA topoisomerase 2-alpha.

## References

[B1] Ablasser A. Goldeck M. Cavlar T. Deimling T. Wittee G. Röhl I.. (2013). cGAS produces a 2’-5’-linked cyclic dinucleotide second messenger that activates STING. Nature 498, 380–384. doi: 10.1038/nature12306 23722158 PMC4143541

[B2] Adefolaju G. A. Theron K. E. Hosie M. J. (2014). Effects of HIV protease, nucleoside/non-nucleoside reverse transcriptase inhibitors on bax, bcl-2 and apoptosis in two cervical cell lines. Biomed. Pharmacother. 68, 241–251. doi: 10.1016/j.biopha.2013.08.007 24011602

[B3] Ahn J. Gutman D. Saijo S. Barber G. N. (2012). STING manifests self DNA-dependent inflammatory disease. Proc. Natl. Acad. Sci. U. S. A. 109, 19386–19391. doi: 10.1073/pnas.1215006109 23132945 PMC3511090

[B4] Anderson S. Bankier A. T. Barrell B. G. de Bruijn M. H. Coulson A. R. Drouin J.. (1981). Sequence and organization of the human mitochondrial genome. Nature 290, 457–465. doi: 10.1038/290457a0 7219534

[B5] Ashour M. E. Atteya R. El-Khamisy S. F. (2015). Topoisomerase-mediated chromosomal break repair: An emerging player in many games. Nat. Rev. Cancer 15, 137–151. doi: 10.1038/nrc3892 25693836

[B6] Barshad G. Marom S. Cohen T. Mishmar D. (2018). Mitochondrial DNA transcription and its regulation: An evolutionary perspective. Trends Genet. 34, 682–692. doi: 10.1016/j.tig.2018.05.009 29945721

[B7] Bersuker K. Hendricks J. M. Li Z. Magtanong L. Ford B. Tang P. H.. (2019). The CoQ oxidoreductase FSP1 acts parallel to GPX4 to inhibit ferroptosis. Nature 575, 688–692. doi: 10.1038/s41586-019-1705-2 31634900 PMC6883167

[B8] Boatright K. M. Salvesen G. S. (2003). Mechanisms of caspase activation. Curr. Opin. Cell Biol. 15, 725–731. doi: 10.1016/j.ceb.2003.10.009 14644197

[B9] Böttinger L. Oeljeklaus S. Guiard B. Rospert S. Warscheid B. Becker T. (2015). Mitochondrial heat shock protein (Hsp) 70 and Hsp10 cooperate in the formation of Hsp60 complexes. J. Biol. Chem. 290, 11611–11622. doi: 10.1074/jbc.M115.642017 25792736 PMC4416864

[B10] Boulares A. H. Yakovlev A. G. Ivanova V. Stoica B. A. Wang G. Iyer S.. (1999). Role of poly(ADP-ribose) polymerase (PARP) cleavage in apoptosis. caspase 3-resistant PARP mutant increases rates of apoptosis in transfected cells. J. Biol. Chem. 274, 22932–22940. doi: 10.1074/jbc.274.33.22932 10438458

[B11] Cai X. Chiu Y. H. Chen Z. J. (2014). The cGAS-cGAMP-STING pathway of cytosolic DNA sensing and signaling. Mol. Cell 54, 289–296. doi: 10.1016/j.molcel.2014.03.040 24766893

[B12] Cao D. Zhao J. Nguyan L. N. Nguyen L. Khanal S. Dang X.. (2019). Disruption of telomere integrity and DNA repair machineries by KML001 induces T cell senescence, apoptosis, and cellular dysfunctions. Front. Immunol. 10, 1152. doi: 10.3389/fimmu.2019.01152 31191531 PMC6540964

[B13] Cao D. Khanal S. Wang L. Li Z. Zhao J. Nguyen L. N.. (2022). A matter of life or death: Productively infected and bystander CD4 T cells in early HIV infection. Front. Immunol. 13, 937057. doi: 10.3389/fimmu.2022.937057 35958582 PMC9361773

[B14] Capranico G. Ferri F. Fogli M. V. Russo A. Lotito L. Baranello L. (2007). The effects of camptothecin on RNA polymerase II transcription: roles of DNA topoisomerase I. Biochimie 89, 482–489. doi: 10.1016/j.biochi.2007.01.001 17336444

[B15] Champoux J. J. (2001). DNA Topoisomerases: Structure, function, and mechanism. Annu. Rev. Biochem. 70, 369–413. doi: 10.1146/annurev.biochem.70.1.369 11395412

[B16] Chen Q. Sun L. Chen Z. J. (2016). Regulation and function of the cGAS-STING pathway of cytosolic DNA sensing. Nat. Immunol 17 (10), 1142–1149. doi: 10.1038/ni.3558 27648547

[B17] Dalla Rosa I. Huang S. Y. Agama K. Khiati S. Zhang H. Pommier Y. (2014). Mapping topoisomerase sites in mitochondrial DNA with a poisonous mitochondrial topoisomerase I (Top1mt). J. Biol. Chem 289 (26), 18595–18602. doi: 10.1074/jbc.M114.555367 24798329 PMC4140277

[B18] Dang X. Ogbu S. C. Zhao J. Nguyen L. Cao D. Nguyen L. N. (2020). Inhibition of topoisomerase IIA (Top2α) induces telomeric DNA damage and T cell dysfunction during chronic viral infection. Cell Death Dis. 11 (3), 196. doi: 10.1038/s41419-020-2395-2 32193368 PMC7081277

[B19] Daroui P. Desai S. D. Li T. K. Liu A. A. Liu L. F. (2004). Hydrogen peroxide induces topoisomerase I-mediated DNA damage and cell death. J. Biol. Chem. 279, 14587–14594. doi: 10.1074/jbc.M311370200 14688260

[B20] Das B. B. Huang S. Y. Murai J. Rehman I. Amé J. C. Sengupta S.. (2014). PARP1-TDP1 coupling for the repair of topoisomerase I-induced DNA damage. Nucleic Acids Res. 42, 4435–4449. doi: 10.1093/nar/gku088 24493735 PMC3985661

[B21] De la loza Díaz M. C. Wellinger R. E. (2009). A novel approach for organelle-specific DNA damage targeting reveals different susceptibility of mitochondrial DNA to the anticancer drugs camptothecin and topotecan. Nucleic Acids Res. 37 (4), e26. doi: 10.1093/nar/gkn1087 19151088 PMC2651790

[B22] Desai S. D. Zhang H. Rodriguez-Bauman A. Yang J. M. Wu X. Gounder M. K.. (2003). Transcription-dependent degradation of topoisomerase I-DNA covalent complexes. Mol. Cell. Biol. 23, 2341–2350. doi: 10.1128/MCB.23.7.2341-2350.2003 12640119 PMC150741

[B23] Doitsh G. Galloway N. L. Geng X. Yang Z. Monroe K. M. Zepeda O.. (2014). Cell death by pyroptosis drives CD4 T-cell depletion in HIV-1 infection. Nature 505, 509–514. doi: 10.1038/nature12940 24356306 PMC4047036

[B24] Doitsh G. Greene W. C. (2016). Dissecting how CD4 T cells are lost during HIV infection. Cell Host Microbe 19, 280–291. doi: 10.1016/j.chom.2016.02.012 26962940 PMC4835240

[B25] Doll S. Freitas F. P. Shah R. Aldrovandi M. da Silva M. C. Ingold I.. (2019). FSP1 is a glutathione-independent ferroptosis suppressor. Nature 575, 693–698. doi: 10.1038/s41586-019-1707-0 31634899

[B26] El-Khamisy S. F. Saifi G. M. Weinfeld M. Johansson F. Helleday T. Lupski J. R.. (2005). Defective DNA single-strand break repair in spinocerebellar ataxia with axonal neuropathy-1. Nature 434, 108–113. doi: 10.1038/nature03314 15744309

[B27] Festjens N. Vanden Berghe T. Cornelis S. Vandenabeele P. (2007). RIP1, a kinase on the crossroads of a cell’s decision to live or die. Cell Death Differ. 14, 400–410. doi: 10.1038/sj.cdd.4402085 17301840

[B28] Fink S. L. Cookson B. T. (2005). Apoptosis, pyroptosis, and necrosis: mechanistic description of dead and dying eukaryotic cells. Infect. Immun. 73, 1907–1916. doi: 10.1128/IAI.73.4.1907-1916.2005 15784530 PMC1087413

[B29] Fouquerel E. Barnes R. P. Uttam S. Watkins S. C. Bruchez M. P. Opresko P.L. (2019). Targeted and persistent 8-oxoguanine base damage at telomeres promotes telomere loss and crisis. Mol. Cell 75, 117–130.e6. doi: 10.1016/j.molcel.2019.04.024 31101499 PMC6625854

[B30] Goswami A. V. Chittoor B. D’Silva P. (2010). Understanding the functional interplay between mammalian mitochondrial Hsp70 chaperone machine components. J. Biol. Chem. 285, 19472–19482. doi: 10.1074/jbc.M110.105957 20392697 PMC2885226

[B31] Guoa D. Y. Dexheimer T. S. Pommier Y. Nasha H. A. (2014). Neuroprotection and repair of 3’-blocking DNA ends by glaikit (gkt) encoding drosophila tyrosyl-DNA phosphodiesterase 1 (TDP1). Proc. Natl. Acad. Sci. U. S. A. 111, 15816–15820. doi: 10.1073/pnas.1415011111 25331878 PMC4226126

[B32] Guo Z. Kozlov S. Lavin M. F. Person M. D. Paull T. T. (2010). ATM Activation by oxidative stress. Science 330, 517–521. doi: 10.1126/science.1192912 20966255

[B33] He J. Wang Y. Missinato M. A. Onuoha E. Perkins L. A. Watkins S. C. (2016). A genetically targetable near-infrared photosensitizer. Nat. Methods 13, 263–268. doi: 10.1038/nmeth.3735 26808669 PMC4916159

[B34] Ji Y. Dang X. Nguyen L. Nguyen L. N. Zhao J. Cao D.. (2019). Topological DNA damage, telomere attrition and T cell senescence during chronic viral infections. Immun. Ageing 16, 1–15. doi: 10.1186/s12979-019-0153-z 31285747 PMC6591813

[B35] Khanal S. Tang Q. Cao D. Zhao J. Nguyen L. N. Oyedeji O. S.. (2020). Telomere and ATM dynamics in CD4 T-cell depletion in active and virus-suppressed HIV infections. J. Virol. 94 (22), e01061–20. doi: 10.1128/JVI.01061-20 32907975 PMC7592222

[B36] Khanal S. Schank M. Gazzar M. El Moorman J. P. Yao Z. Q. (2021). HIV-1 latency and viral reservoirs: Existing reversal approaches and potential technologies, targets, and pathways involved in HIV latency studies. Cells 10, 1–23. doi: 10.3390/cells10020475 PMC7926981 33672138

[B37] Kuo H. H. Ahmad R. Lee G. Q. Gao C. Chen H. R. Ouyang Z. (2018). Anti-apoptotic protein BIRC5 maintains survival of HIV-1-Infected CD4 + T cells. Immunity 48 (6), 1183–1194.e5. doi: 10.1016/j.immuni.2018.04.004 29802019 PMC6013384

[B38] Li T. Chen Z. J. (2018). The cGAS-cGAMP-STING pathway connects DNA damage to inflammation, senescence, and cancer. J. Exp. Med. 215, 1287–1299. doi: 10.1084/jem.20180139 29622565 PMC5940270

[B39] Li G. Y. Zhou Y. Ying R. S. Shi L. Cheng Y. Q. Ren J. P.. (2015). Hepatitis c virus-induced reduction in miR-181a impairs CD4+ T-cell responses through overexpression of DUSP6. Hepatology 61, 1163–1173. doi: 10.1002/hep.27634 25477247 PMC4376593

[B40] Liu S. Cai X. Wu J. Cong Q. Chen X. Li T.. (2015). Phosphorylation of innate immune adaptor proteins MAVS, STING, and TRIF induces IRF3 activation. Science 347 (6227), aaa2630. doi: 10.1126/science.aaa2630 25636800

[B41] Miao Z. H.. (2006). Hereditary ataxia SCAN1 cells are defective for the repair of transcription-dependent topoisomerase I cleavage complexes. DNA Repair (Amst). 5, 1489–1494. doi: 10.1016/j.dnarep.2006.07.004 16935573

[B42] Nguyen L. N. Zhao J. Cao D. Dang X. Wang L. Lian J.. (2018). Inhibition of TRF2 accelerates telomere attrition and DNA damage in naïve CD4 T cells during HCV infection. Cell Death Dis. 9 (9), 900. doi: 10.1038/s41419-018-0897-y 30185784 PMC6125360

[B43] Nguyen L. N. Nguyen L. Zhao J. Schank M. Dang X. Cao D.. (2021). Immune activation induces telomeric DNA damage and promotes short-lived effector T cell differentiation in chronic HCV infection. Hepatology 74 (5), 2380–2394. doi: 10.1002/HEP.32008 34110660 PMC8542603

[B44] Nguyen L. Nguyen L. N. Zhao J. Schank M. Dang X. Cao D.. (2021). Long non-coding RNA GAS5 regulates T cell functions *via* miR21-mediated signaling in people living with HIV. Front. Immunol. 12, 601298. doi: 10.3389/fimmu.2021.601298 33776993 PMC7994762

[B45] Patel A. G.. (2016). Immunodetection of human topoisomerase I-DNA covalent complexes. Nucleic Acids Res. 44, 2816–2826. doi: 10.1093/nar/gkw109 26917015 PMC4824114

[B46] Pommier Y. (2006). Topoisomerase I inhibitors: Camptothecins and beyond. Nat. Rev. Cancer 6 (10), 789–802. doi: 10.1038/nrc1977 16990856

[B47] Pommier Y. (2013). Drugging topoisomerases: Lessons and challenges. ACS Chem. Biol. 8, 82–95. doi: 10.1021/cb300648v 23259582 PMC3549721

[B48] Pommier Y. Barcelo J. M. Rao V. A. Sordet O. Jobson A. G. Thibaut L.. (2006). Repair of topoisomerase I-mediated DNA damage. Prog. Nucleic Acid Res. Mol. Biol 81, 179–229. doi: 10.1016/S0079-6603(06)81005-6 16891172 PMC2576451

[B49] Pommier Y. Leo E. Zhang H. Marchand C. (2010). DNA Topoisomerases and their poisoning by anticancer and antibacterial drugs. Chem. Biol. 17, 421–433. doi: 10.1016/j.chembiol.2010.04.012 20534341 PMC7316379

[B50] Pouliot J. J. Yao K. C. Robertson C. A. Nash H. A. (1999). Yeast gene for a tyr-DNA phosphodiesterase that repairs topoisomerase I complexes. Science 286 (5439), 552–555. doi: 10.1126/science.286.5439.552 10521354

[B51] Rooney J. P.. (2015). PCR based determination of mitochondrial DNA copy number in multiple species. Methods Mol. Biol. 1241, 23–38. doi: 10.1007/978-1-4939-1875-1_3 25308485 PMC4312664

[B52] Schank M. Zhao J. Wang L. Li Z. Cao D. Nguyen L. N.. (2020). Telomeric injury by KML001 in human T cells induces mitochondrial dysfunction through the p53-PGC-1α pathway. Cell Death Dis. 11 (12), 1030. doi: 10.1038/s41419-020-03238-7 33268822 PMC7710715

[B53] Schank M.. (2021). Oxidative stress induces mitochondrial compromise in CD4 T cells from chronically HCV-infected individuals. Front. Immunol. 12. doi: 10.3389/fimmu.2021.760707 PMC8692574 34956192

[B54] Schank M. Zhao J. Moorman J. P. Yao Z. Q. (2021). The impact of HIV- and ART-induced mitochondrial dysfunction in cellular senescence and aging. Cells 10, 1–21. doi: 10.3390/cells10010174 PMC7830696 33467074

[B55] Scheid M. P. Schubert K. M. Duronio V. (1999). Regulation of bad phosphorylation and association with bcl-x(L) by the MAPK/Erk kinase. J. Biol. Chem. 274, 31108–31113. doi: 10.1074/jbc.274.43.31108 10521512

[B56] Shi L.. (2014). KLRG1 impairs CD4+ T cell responses *via* p16ink4a and p27kip1 pathways: role in hepatitis b vaccine failure in individuals with hepatitis c virus infection. J. Immunol. 192, 649–657. doi: 10.4049/jimmunol.1302069 24337749 PMC3894750

[B57] Stockwell B. R.. (2017). Ferroptosis: A regulated cell death nexus linking metabolism, redox biology, and disease. Cell 171, 273–285. doi: 10.1016/j.cell.2017.09.021 28985560 PMC5685180

[B58] Telmer C. A.. (2015). Rapid, specific, no-wash, far-red fluorogen activation in subcellular compartments by targeted fluorogen activating proteins. ACS Chem. Biol. 10, 1239–1246. doi: 10.1021/cb500957k 25650487 PMC4867890

[B59] Vos S. M. Tretter E. M. Schmidt B. H. Berger J. M. (2011). All tangled up: How cells direct, manage and exploit topoisomerase function. Nat. Rev. Mol. Cell Biol. 12, 827–841. doi: 10.1038/nrm3228 22108601 PMC4351964

[B60] Wang J. C. (2002). Cellular roles of DNA topoisomerases: A molecular perspective. Nat. Rev. Mol. Cell Biol. 3, 430–440. doi: 10.1038/nrm831 12042765

[B61] Wang L. Lu Z. Zhao J. Schank M. Cao D. Dang X.. (2021). Selective oxidative stress induces dual damage to telomeres and mitochondria in human T cells. Aging Cell 20 (12), e13513. doi: 10.1111/acel.13513 34752684 PMC8672791

[B62] Warren C. F. A. Wong-Brown M. W. Bowden N. A. (2019). BCL-2 family isoforms in apoptosis and cancer. Cell Death Dis. 10 (3), 177. doi: 10.1038/s41419-019-1407-6 30792387 PMC6384907

[B63] Yao Z. Q. Moorman J. P. (2013). Immune exhaustion and immune senescence: Two distinct pathways for HBV vaccine failure during HCV and/or HIV infection. Archivum Immunol. Ther. Experimentalis 61, 193–201. doi: 10.1007/s00005-013-0219-0 PMC3792483 23400275

[B64] Zhao J.. (2018). Insufficiency of DNA repair enzyme ATM promotes naive CD4 T-cell loss in chronic hepatitis c virus infection. Cell Discovery 4, 16. doi: 10.1038/s41421-018-0015-4 29644094 PMC5891503

[B65] Zhao J.. (2019). ATM Deficiency accelerates DNA damage, telomere erosion, and premature T cell aging in HIV-infected individuals on antiretroviral therapy. Front. Immunol. 10, 2531. doi: 10.3389/fimmu.2019.02531 31781094 PMC6856652

[B66] Zhao J.. (2021). Mitochondrial functions are compromised in CD4 T cells from ART-controlled PLHIV. Front. Immunol. 0, 1539. doi: 10.3389/fimmu.2021.658420 PMC8129510 34017335

[B67] Zhou Y.. (2016). Protection of CD4 + T cells from hepatitis c virus infection-associated senescence *via* ΔNp63–miR-181a–Sirt1 pathway. J. Leukoc. Biol. 100, 1201–1211. doi: 10.1189/jlb.5A0316-119RR 27354409 PMC5069086

