# Supplementary figures and images for: Mitochondrial topoisomerase 1 inhibition induces topological DNA damage and T cell dysfunction in patients with chronic viral infection
Source: Front Cell Infect Microbiol. 2022 Nov 3;12:1026293. doi: 10.3389/fcimb.2022.1026293 (PMC9669385; doi:10.3389/fcimb.2022.1026293)

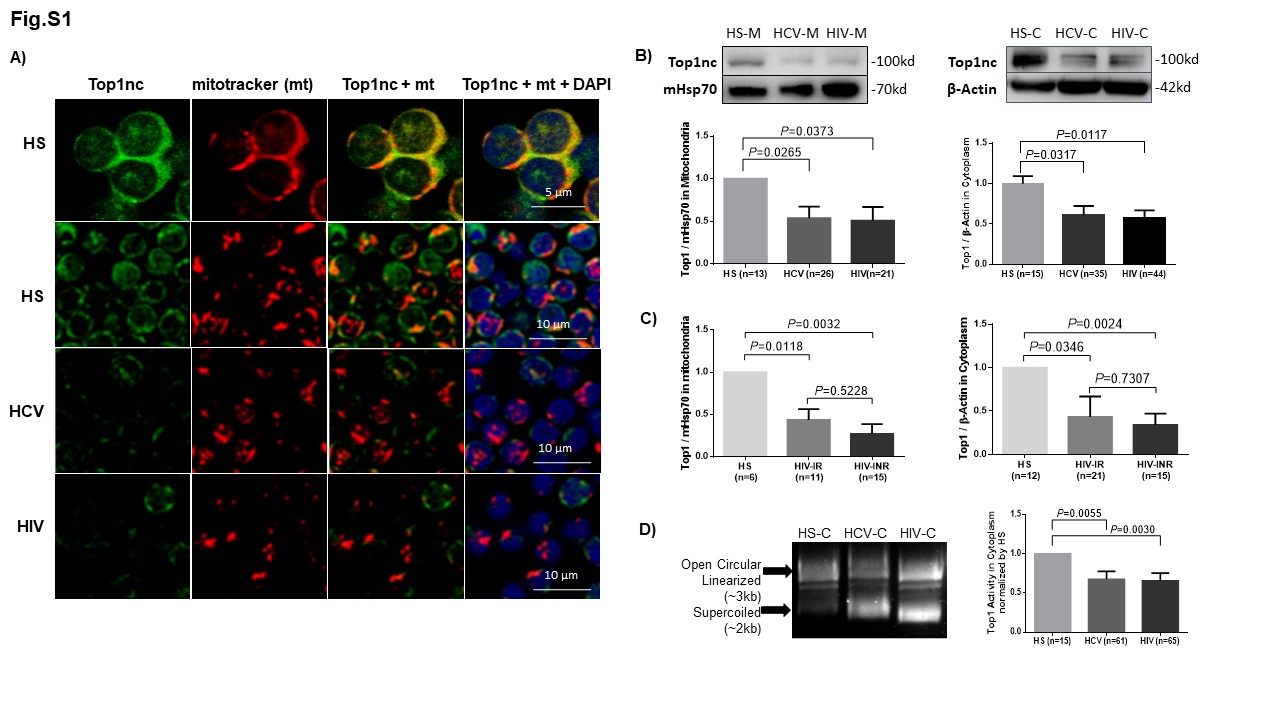

Supplement: Supplementary file 1 [file Image_1.jpeg]

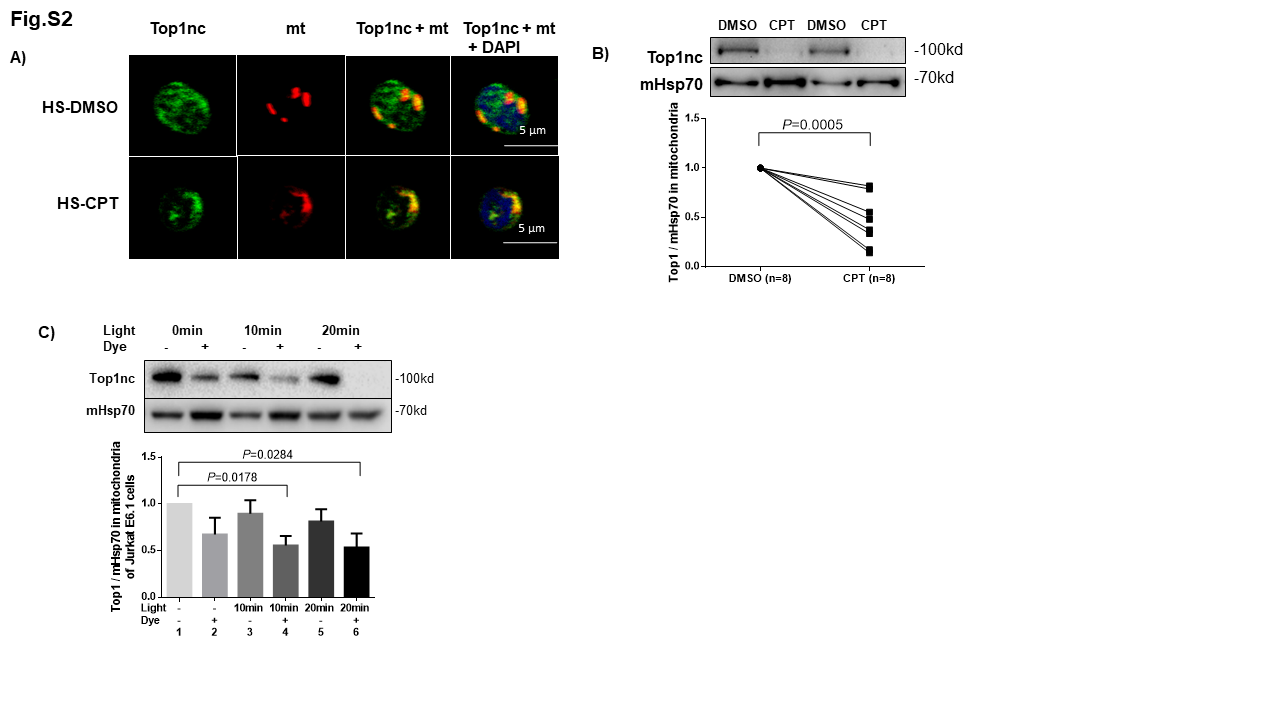

Supplement: Supplementary file 2 [file Image_2.tif]
